# Au(I)/Au(III)-catalyzed Sonogashira-type reactions of functionalized terminal alkynes with arylboronic acids under mild conditions

**DOI:** 10.3762/bjoc.7.92

**Published:** 2011-06-15

**Authors:** Deyun Qian, Junliang Zhang

**Affiliations:** 1 Shanghai Key Laboratory of Green Chemistry and Chemical Processes, Department of Chemistry, East China Normal University, 3663 N, Zhongshan Road, Shanghai 200062 (P. R. China); 2 State Key Laboratory of Organometallic Chemistry, Shanghai Institute of Organic Chemistry, Chinese Academy of Sciences, Ling Ling Road 345 Shanghai 200032 (P. R. China)

**Keywords:** arylboronic acid, gold-catalysis, Sonogashira cross-coupling

## Abstract

A straightforward, efficient, and reliable redox catalyst system for the Au(I)/Au(III)-catalyzed Sonogashira cross-coupling reaction of functionalized terminal alkynes with arylboronic acids under mild conditions has been developed.

## Introduction

The Sonogashira reaction has become the most important and widely used method for the synthesis of arylalkynes and conjugated enynes, which are precursors for natural products, pharmaceuticals and other materials [1–3]. In the past decade, considerable efforts have been made to enhance the efficiency and generality of this reaction. All kinds of palladium catalyst systems [4–10] and other metal catalyst systems [3] have been developed for facilitating the Sonogashira cross-coupling, as well as expanding the substrate scope [11–14]. Various examples have recently been reported for the palladium-catalyzed Sonagashira-type cross-coupling of terminal alkynes with arylboronic acids [13], and the Pd(0)/Pd(II) catalytic cycles have been well studied. Nevertheless, this transformation catalyzed by gold, involving Au(I)/Au(III) catalytic cycles has, as yet, been less explored [15–22]. In the few examples already documented some conditions, such as rather high reaction temperatures (130 °C), high catalysis loading or special reagents were required [23]. Herein, we report a straightforward, efficient and robust catalyst system for the Sonogashira-type cross-coupling, in which Au(I)/Au(III) catalyzed C_sp_^2^–C_sp_ bond formation of terminal alkynes from arylboronic acids under mild conditions.

**Scheme 1:**
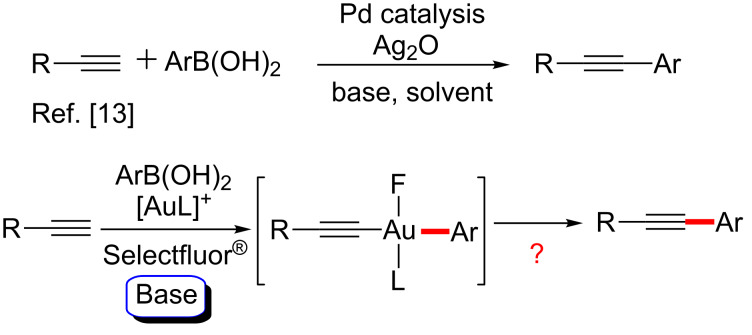
Previous work and our projected gold-catalyzed Sonogashira-type cross-coupling.

By analogy with other d^10^ species, Au(I) has the same electronic structure as Pd(0) and can easily interact with the acetylenic group, and has the ability to undergo the Au(I)/Au(III) redox cycles [24–25]. In addition, with an increasing interest in the chemistry of gold(I) and gold(III) compounds, more and more studies have provided strong evidence for the existence of Au(I)/Au(III) catalytic cycles [26–32]. For instance, Zhang and co-workers have developed a gold-catalyzed oxidative cross-coupling reaction of arylboronic acids with propargyl esters [27], and Selectfluor^®^ – a source of electrophilic fluorine – was used to oxidize the resulting Au(I) intermediate to Au(III) species. Recently, Toste reported the first experimental evidence for alkylgold(III) fluorides undergoing C–C bond forming reactions with arylboronic acids [32]. Inspired by these results, we envisioned that terminal alkynes would react with arylboronic acids in the presence of oxidant (Selectfluor^®^) and base, and undergo a Au(I)/Au(III)-catalyzed Sonogashira-type cross-coupling reaction (Scheme 1).

## Results and Discussion

We embarked on developing a general protocol for Sonogashira-type cross-coupling by using propargyl tosylamide (**1a**) and phenylboronic acid as the model substrates because of their stability, availability, and broad spectrum of nucleophilicity (Table 1). With commercially available Ph_3_PAuCl as the catalyst, we treated **1a** and phenylboronic acid in CH_3_CN at room temperature for 18 h (Table 1, entry 1). Disappointedly, only trace amounts of product was observed under the above conditions. Taking into account the counter ion effects in gold-catalyzed reactions [33–35], Ph_3_PAuOTf, produced from a combination of Ph_3_PAuCl and AgOTf in a 1:1 ratio, was investigated. To our delight, the expected product **2a** could be isolated in 56% yield (Table 1, entry 2). Moreover, a control experiment was carried out to determine whether AgOTf by itself could catalyze this transformation. However, no **2a** was produced in the absence of Ph_3_PAuCl (Table 1, entry 3). Without the phosphine ligand, both AuCl/AgOTf or AuCl_3_/AgOTf could catalyze this reaction, but the efficiency was quite low (Table 1, entries 4, 5). Without Selectfluor^®^ as additive, the cross-coupling reaction did not occur (Table 1, entry 6), indicating that it was crucial for this transformation via oxidation of gold(I) to the gold(III) species [26–32]. Reducing the amount of phenylboronic acid resulted in a lower yield (Table 1, entry 7). The counter ion effect was then examined on cross-coupling reaction by variation of the silver salts. Alternative catalyst systems led to better yields (Table 1, entries 8, 9 and notably 11 vs entry 2). To optimize the reaction conditions, commonly employed well recognized and commercially available phosphine ligands [17–18], such as dppm, dppe, dppp, dppf, and XPhos, were screened to test the feasibility of this gold-catalyzed cross-coupling (Table 1, entries 10–12, see also Supporting Information File 1). In addition, a series of inorganic and organic bases was also investigated: K_2_CO_3_, K_3_PO_4_, K_3_PO_4_·3H_2_O, NaHCO_3_, NaOAc were substantially less effective, whilst organic bases such as iPr_2_NH, Et_2_NH, TMEDA, Bu_3_N, PhNMe_2_ were not effective bases in this catalytic system (see Supporting Information File 1). These results indicated that Et_3_N might also play an important role in this process. Furthermore, it was also found that neither slow addition nor a significant excess of alkyne was required to obtain selective and almost quantitative conversion (see Supporting Information File 1). When the reaction was carried out under an atmosphere of nitrogen (N_2_) there was a 10% increase in yield (Table 1, entry 8 vs 14). Although the conditions (Table 1, entry 11) seemed the best, this was not the general case for other substrates. Therefore, the optimum conditions were chosen as Ph_3_PAuCl/AgBF_4_ as the catalyst, Et_3_N as the base and under an atmosphere of nitrogen (Table 1, entry 14) .

**Table 1:** Initial screenings of Au(I)/Au(III)-catalyzed Sonogashira coupling^a^.

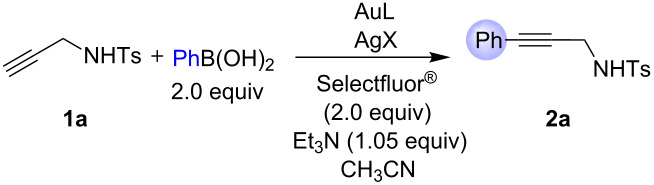				

Entry	AuL (5 mol %)	AgX (mol %)	Time (h)	Yield (%)^b^

1	Ph_3_PAuCl	—	18	trace
2	Ph_3_PAuCl	AgOTf (5)	22	56
3	—	AgOTf (5)	24	0
4	AuCl	AgOTf (5)	24	21
5	AuCl_3_	AgOTf (15)	24	37^c^
6^d^	Ph_3_PAuCl	AgOTf (5)	22	0
7^e^	Ph_3_PAuCl	AgOTf (5)	22	41
8	Ph_3_PAuCl	AgBF_4_ (5)	10	65
9	Ph_3_PAuCl	AgSbF_4_(5)	10	62
10	(XPhos)AuCl	AgOTf (5)	24	0
11^f^	dppm(AuCl)_2_	AgOTf (5)	16	83^c^
12^f^	dppm(AuBr)_2_	—	16	trace
13^f,g^	Ph_3_PAuCl	AgOTf (5)	12	72 (73^c^)
14^f^	Ph_3_PAuCl	AgBF_4_ (5)	12	75 (80^c^)

^a^Reaction conditions: The reaction was carried out by using **1a** (0.4 mmol) and phenylboronic acid (0.8 mmol, 2.0 equiv), and 1.05 equiv of Et_3_N in 2 mL of CH_3_CN stirred at room temperature. ^b^Isolated yields. ^c^Yield determined by ^1^H NMR with dibromomethane as an internal standard. ^d^Selectfluor^®^ (0 equiv). ^e^PhB(OH)_2_ (1.5 equiv). ^f^Under an atmosphere of nitrogen (N_2_). ^g^The reaction temperature was 50 °C.

**Scheme 2:**
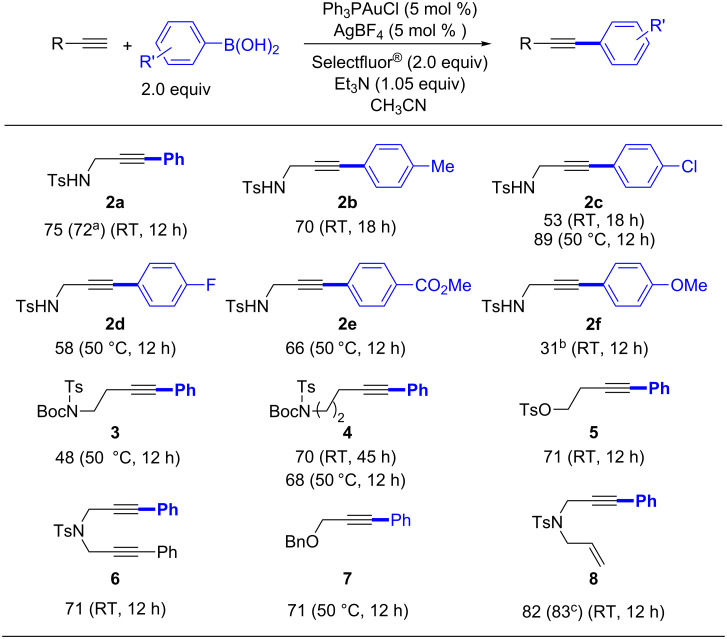
Scope of the Sonogashira-type cross-coupling reaction (isolated yield). ^a^AgOTf in place of AgBF_4_. ^b^Yield determined by ^1^H NMR, the prouduct could not be separated from the unreacted starting material. ^c^AgOTf in place of AgBF_4_, RT, 36 h.

By using the above optimized conditions, the reaction scope was next studied by varying arylboronic acids. As shown in Scheme 2, functional groups including methyl, chloro, fluoro and ester in the para positions of the phenyl ring were tolerated. Reduced yields were observed with both electron-rich and electron-poor coupling partners (Scheme 2, **2a**–**f**). Arylboronic acids with electron-withdrawing groups afforded **2c**–**2e** in moderate to good yields. Notably, the slightly electron-deficient 4-chlorophenylboronic acid gave the best yield. On the other hand, the more electron-rich 4-methoxyphenylboronic acid produced the lowest yield, likely due to a competing oxidation of the boronic acid by Selectfluor^®^ [27,30]. Gratifyingly, a number of potentially reactive functionalities, such as tertiary amine, 4-methylbenzenesulfonate, 1,6-enyne and 1,6-diyne, were compatible and remained unaffected, which illustrated the robustness of the catalyst system (Scheme 2, **3**–**8**).

**Scheme 3:**
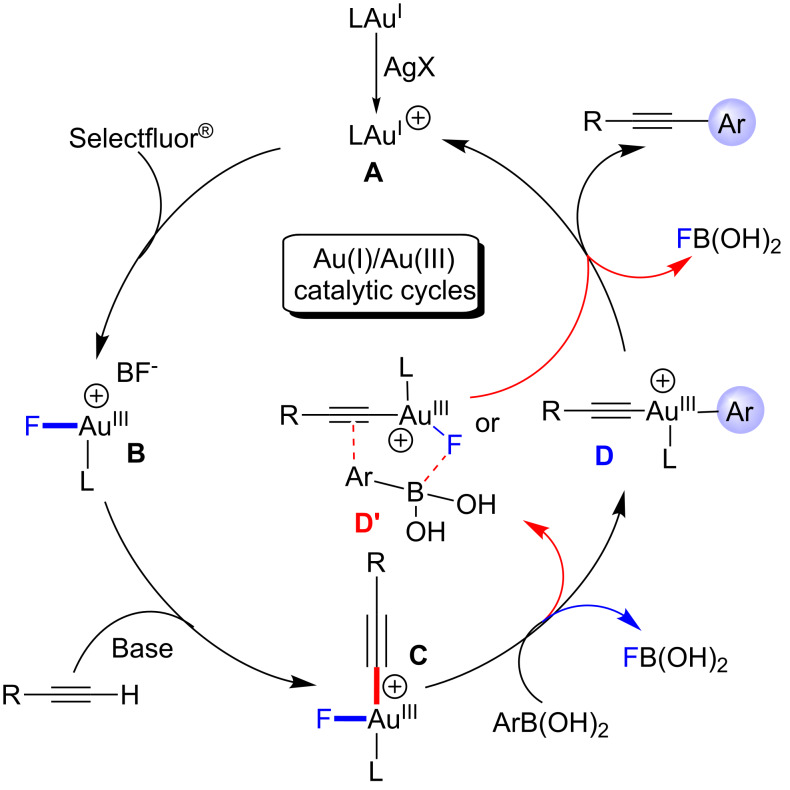
Proposed mechanism for the Au(I)/Au(III)-catalyzed Sonogashira-type cross-coupling.

On the basis of these results, a plausible mechanism is outlined in Scheme 3. Initially, unlike traditional C–X oxidative addition as shown by Pd in cross-coupling reactions, the active cationic gold(I) species **A** is oxidized by Selectfluor^®^ to give a gold(III) species **B** [26–32,36]. With the aid of base, the reaction between **B** and alkyne affords intermediate **C**. The weak Au–F bond and the strong B–F bond drive the trans-metalation to produce intermediate **D** [29,36–37]. Finally, **D** undergoes reductive elimination to give the desired product and gold(I) species **A**. In addition, **C** also could experience a five-centered transition state **D'**, which leads to the C–C bond-forming reaction through a bimolecular reductive elimination [30–32]. Notably, we believed that the key step of this mechanism is the generation of cationic gold species **B** by Selectfluor^®^. In the absence of Selectfluor^®^, no coupling is possible (Table 1, entry 6). In a current study, Xu and co-workers have provided strong evidence for the oxidation of Au(I) to Au(III) by Selectfluor^®^ in their XPS measurements [36].

## Conclusion

In conclusion, we have developed an unprecedented Au(I)/Au(III)-catalyzed Sonogashira-type cross-coupling reaction of terminal alkynes and arylboronic acids under mild conditions. Selectfluor^®^ and counter ion effects play a significant role in the development of an exceptionally mild catalyst system. This chemistry strongly suggests the feasibility of Au(I) and Au(III) catalytic redox cycles, which would substantially broaden the field of gold catalysis and offer more functionalized products. Furthermore, the good tolerance toward many functional groups of substrates considerably extends the scope of a number of organic transformations and performs modular C_sp_^2^–C_sp_ bond constructions at appropriate stages in the whole synthetic sequence.

## Supporting Information

Supporting information features experimental procedure and spectroscopic data.

File 1 Experimental details and spectra of new compounds.
