# Supplementary material for: Au(I)/Au(III)-catalyzed Sonogashira-type reactions of functionalized terminal alkynes with arylboronic acids under mild conditions
Source: Beilstein J Org Chem. 2011 Jun 15;7:808–12. doi: 10.3762/bjoc.7.92 (PMC3135097; doi:10.3762/bjoc.7.92)
Supplement: File 1 — Experimental details and spectra of new compounds. [file Beilstein_J_Org_Chem-07-808-s001.pdf]

Supporting Information

for

Au(I)/Au(III)-catalyzed Sonogashira-type reactions of functionalized terminal alkynes with arylboronic acids under mild conditions

Deyun Qian[1] and Junliang Zhang *[1,2]

Address: [1]Shanghai Key Laboratory of Green Chemistry and Chemical Processes, Department of Chemistry, East China Normal University, 3663 N, Zhongshan Road, Shanghai 200062 (P. R. China) and [2]State Key Laboratory of Organometallic Chemistry, Shanghai Institute of Organic Chemistry, Chinese Academy of Sciences, Ling Ling Road 345 Shanghai 200032 (P. R. China)

E-mail: Deyun Qian - 51100606057@ecnu.cn; Junliang Zhang - jlzhang@chem.ecnu.edu.cn

* Corresponding author

Experimental details and spectra of new compounds

Content	Page number
**1. General information**	**S2**
**2. General procedure for conditions screening**	**S3**
**3. General procedure**	**S6**
**4. References**	**S12**
**5. ^{1}H and ^{13}C NMR spectra**	**S13**

1. General information

Unless otherwise noted, commercial materials were directly used without further purification. Anhydrous acetonitrile (MeCN) and triethylamine (NEt_3) were distilled from CaH_2 under a nitrogen atmosphere prior to use. Reactions were monitored by thin layer chromatography (TLC) using Whatman® pre-coated silica gel plates. Flash column chromatography was performed on SiliCycle® silica gel (230–400 mesh). ^{1}H NMR and ^{13}C NMR spectra were recorded with a Bruker 400 MHz spectrometer and chemical shifts are reported in ppm, relative to $CHCl_3$ (7.26 ppm for ^{1}H, and 77.00 ppm for ^{13}C) unless otherwise noted. Splitting patterns of an apparent multiplet associated with an averaged coupling constant were designed as s (singlet), d (doublet), t (triplet), q (quartet), and m (multiplet).

2. General procedure for conditions screening

Propargyl tosylamide **1a** (83.7 mg, 0.4 mmol) was dissolved in MeCN (2 mL) and $PhB(OH)_2$ (97.5 mg, 0.8 mmol), the gold catalyst (5 mol % or 2.5 mol %) and base (1.05 equiv) added followed by Selectfluor® (283.4 mg, 0.8 mmol). The reaction mixture was stirred at room temperature for 10–24 h and the reaction was monitored by TLC. After completion, the reaction mixture was diluted with EtOAc and filtered through a pad of celite. The filtrate was concentrated under reduced pressure and the crude product purified by chromatography on silica gel (using hexanes/EtOAc as eluent).

Table S1:

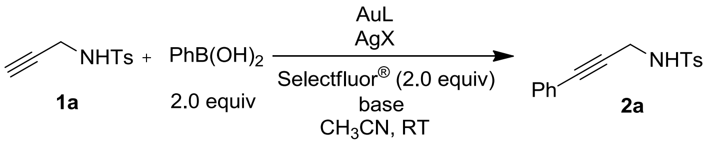

entry	AuL (mol %)	AgX (mol %)	base (equiv)	t (h)	Yield (%)
**1**	Ph_3PAuCl (5)	-	Et_3N (1.05)	22	trace
**2**	Ph_3PAuCl (5)	AgOTf (5)	Et_3N (1.05)	18	56
**3**	AuCl (5)	AgOTf (5)	Et_3N (1.05)	24	21
**4**	-	AgOTf (5)	Et_3N (1.05)	24	0
**5**[b]	Ph_3PAuCl (5)	AgOTf (5)	Et_3N (1.05)	22	0
**6**[c]	Ph_3PAuCl (5)	AgOTf (5)	Et_3N (1.05)	22	41
**7**	Ph_3PAuCl (5)	$AgBF_4$ (5)	Et_3N (1.05)	10	65

**8**	Ph_3PAuCl (5)	$AgSbF_4$(5)	Et_3N (1.05)	10	62
**9**	Ph_3PAuCl (5)	$AgNO_3$(5)	Et_3N (1.05)	10	45[a]
**10**	Ph_3PAuCl (5)	$AgPF_6$(5)	Et_3N (1.05)	10	40[a]
**11**	Ph_3PAuCl (5)	AgOAc(5)	Et_3N (1.05)	10	0
**12**	Ph_3PAuCl (5)	$AgBF_4$ (5)	Et_3N (1.2)	10	69[a]
**13**	dppf$(AuCl)_2$ (2.5)	AgOTf (5)	Et_3N (1.05)	14	39[a]
**14**	dppp$(AuCl)_2$ (2.5)	AgOTf (5)	Et_3N (1.05)	14	56 (65[a])
**15**	dppp$(AuCl)_2$ (2.5)	$AgBF_4$ (5)	Et_3N (1.05)	14	0
**16**	dppe$(AuCl)_2$ (2.5)	AgOTf (5)	Et_3N (1.05)	14	48[a]
**17**	dppb$(AuCl)_2$ (2.5)	AgOTf (5)	Et_3N (1.05)	14	65[a]
**18**	dppp$(AuCl)_2$ (5)	AgOTf (5)	Et_3N (1.05)	16	59[a]
**19**	dppp$(AuCl)_2$ (5)	AgOTf (5)	$K_3PO_4 \cdot 3H_2O$ (1.5)	22	59
**20**	dppp$(AuCl)_2$ (5)	AgOTf (5)	$K_3PO_4 \cdot 3H_2O$ (1.5)+ Et_3N (0.5)	22	47
**21**	dppp$(AuCl)_2$ (5)	AgOTf (5)	K_2CO_3 (1.5)	22	34
**22**	Ph_3PAuCl (5)	AgOTf (5)	Et_3N (1.5)	22	53
**23**	Ph_3PAuCl (5)	AgOTf (5)	iPr_2NH (1.05)	12	trace
**24**	Ph_3PAuCl (5)	AgOTf (5)	Bu_3N (1.05)	12	trace
**25**	Ph_3PAuCl (5)	AgOTf (5)	TMEDA (1.05)	12	trace
**26**	Ph_3PAuCl (5)	AgOTf (5)	$PhNMe_2$ (1.05)	12	ND

[a]Yield determined by ^{1}H NMR. [b]Selectfluor® (0 equiv). [c]$PhB(OH)_2$ (1.5 equiv).

Table S2:

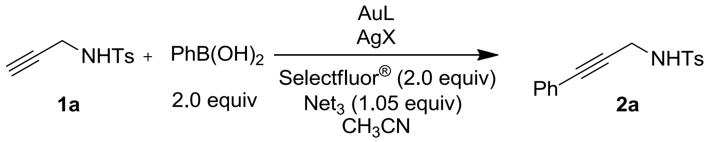

entry	1a (mmol)	AuL (mol %)	AgX (mol %)	T (°C)	t (h)	product (yield %)
**1**	0.4	(XPhos)AuCl (5)	AgOTf (5)	RT	24	trace
**2**[a]	0.4	(XPhos)AuCl (5)	AgOTf (5)	RT	24	trace
**3**	0.4	Ph_3PAuCl (5)	$AgBF_4$ (5)	55	3.5	63
**4**[b]	0.48	Ph_3PAuCl (5)	$AgBF_4$ (5)	RT	24	trace
**5**[c]	0.4	Ph_3PAuCl (5)	$AgBF_4$ (5)	RT	24	trace
**6**[d]	0.48	Ph_3PAuCl (5)	AgOTf (5)	RT	24	ND
**7**	0.4	Ph_3PAuCl (5)	AgOTf (5)	40	24	46

[a]base: $K_3PO_4\cdot3H_2O$ (1.5 equiv). [b]S: $PhB(OH)_2$ = 1.2: 1. [c]**1a**: $PhB(OH)_2$ = 1.5: 1. [d]**1a**: $PhB(OH)_2$ = 1.5: 1.

Table S3[a]:

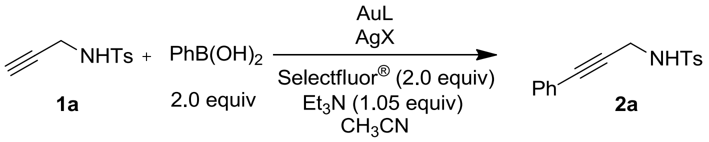

entry	1a (mmol)	AuL (mol %)	AgX (mol %)	T (°C)	t (h)	product (yield %)
**1**	0.4	Ph_3PAuCl (5)	AgOTf (5)	50	12	72 (73[b])
**2**	0.4	Ph_3PAuCl (5)	AgOTf (5)	rt	12	59[b]
**3**	0.2	Ph_3PAuCl (5)	$AgBF_4$ (5)	rt	12	80[b]

**4**	0.2	Ph_3PAuCl (5)	$AgPF_6$ (5)	rt	16	40[b]
**5**	0.2	Ph_3PAuCl (5)	AgOAc (5)	rt	16	0
**6**	0.4	Ph_3PAuCl (5)	$AgBF_4$ (5)	rt	12	75(80[b])
**7**	0.4	Ph_3PAuCl (5)	AgOTf (5)	rt	16	70[b]
**8**	0.2	$dppm(AuCl)_2$ (5)	AgOTf (5)	rt	16	83[b]
**9**	0.4	$dppm(AuBr)_2$ (5)	-	rt	16	trace

[a]The reaction was carried out under an atmosphere of nitrogen (N_2). [b]Yield determined by ^{1}H NMR.

3. General procedure

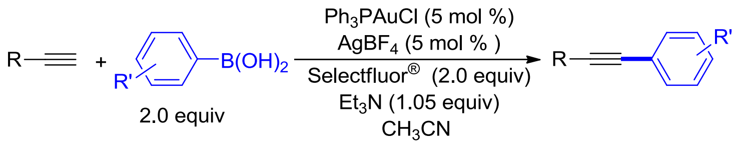

All reactions were carried out under an atmosphere of nitrogen (N_2).

The alkyne (83.7 mg, 0.4 mmol) was dissolved in MeCN (2 mL), $PhB(OH)_2$ (97.5 mg, 0.8 mmol), Ph_3PAuCl (5 mol %), $AgBF_4$ (5 mol %) and Et_3N (1.05 equiv) were added followed by Selectfluor® (283.4 mg, 0.8 mmol). The reaction mixture was stirred at the noted temperature for 12–45 h and the reaction monitored by TLC. After completion, the reaction mixture was diluted with EtOAc and filtered through a pad of celite. The filtrate was concentrated under reduced pressure and the crude product was purified by chromatography on silica gel (using hexanes/EtOAc as eluents).

(1) *N*-(3-Phenylprop-2-ynyl)-*p*-toluenesulfonamide (2a)

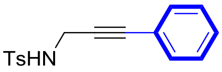

2a

The reaction of propargyl tosylamide **1a** and phenylboronic acid was carried out at room temperature for 12 h to afford compound **2a** (hexanes/EtOAc = 3/1) in 75% yield as a pale orange solid. Its spectroscopic data were in accord with those reported in the literature [1]. ^{1}H NMR (400 MHz, $CDCl_3$) δ 7.81 (d, *J* = 8.0 Hz, 2H), 7.30–7.22 (m, 5H), 7.11 (d, *J* = 8.0 Hz, 2H), 4.88 (t, *J* = 5.6 Hz, 1H), 4.06 (d, *J* = 6.4 Hz, 2H), 2.34 (s, 3H); ^{13}C NMR (100 MHz, $CDCl_3$) δ 143.7, 136.8, 131.5, 129.7, 128.4, 128.1, 127.5, 122.0, 84.7, 83.2, 33.7, 21.4.

(2) 4-Methyl-*N*-(3-*p*-tolylprop-2-ynyl)benzenesulfonamide (2b)

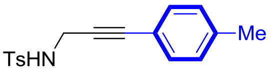

2b

The reaction of propargyl tosylamide **1b** and phenylboronic acid was carried out at room temperature for 18 h to afford compound **2b** (hexanes/EtOAc = 5/1) in 70% yield as a white solid. Its spectroscopic data were in accord with those reported in the literature [2]. ^{1}H NMR (400 MHz, $CDCl_3$) δ 7.80 (d, *J* = 7.6 Hz, 2H), δ 7.26 (d, *J* = 8.0 Hz, 2H), 7.05–7.00 (m, 4H), 4.88 (t, *J* = 6.0 Hz, 1H), 4.06 (d, *J* = 6.0 Hz, 2H), 2.36 (s, 3H), 2.32 (s, 3H); ^{13}C NMR (100 MHz, $CDCl_3$) δ 143.7, 138.6, 136.8, 131.4, 129.6, 128.9, 127.4, 118.9, 84.8, 82.5, 33.8, 21.42, 21.40. EI-MS (*m*/*z*, relative intensity): 299 (M^+, 2.99), 91 (100). HRMS (EI) calcd. for $[C_{17}H_{17}NO_2S]^+$: 299.0980, found 299.0984.

(3) *N*-[3-(4-Chlorophenyl)-prop-2-ynyl]-*p*-toluenesulfonamide (2c)

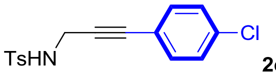

2c

The reaction of propargyl tosylamide **1c** and 4-chlorophenylboronic acid was carried out at 50 °C for 12 h to afford **2c** (hexanes/EtOAc = 5/1) in 89% yield as a white solid. Its spectroscopic data were in accord with those reported in the literature [3]. ^{1}H NMR (400 MHz, CDCl3) δ 7.80 (d, *J* = 8.0 Hz, 2H), 7.26 (d, *J* = 7.6 Hz, 2H), 7.21 (d, *J* = 7.6 Hz, 2H), 7.04 (d, *J* = 8.0 Hz, 2H), 4.85 (t, *J* = 6.0 Hz, 1H), 4.06 (d, *J* = 6.0 Hz, 2H), 2.36 (s, 3H); ^{13}C NMR (100 MHz, $CDCl_3$) δ 143.8, 136.8, 134.6, 132.7, 129.7, 128.5, 127.5, 120.5, 84.2, 83.6, 33.7, 21.5. EI-MS (*m*/*z*, relative intensity): 319 (M^+, 1.89), 164 (100). HRMS (EI) calcd. for $[C_{16}H_{14}NO_2SCl]^+$: 319.0434, found 319.0441.

(4) *N*-[3-(4-Fluorophenyl)-prop-2-ynyl]-*p*-toluenesulfonamide (2d)

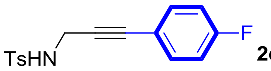

2d

The reaction of propargyl tosylamide **1d** and phenylboronic acid was carried out at 50 °C for 12 h to afford **2d** (hexanes/EtOAc = 5/1) in 58% yield as a white solid. ^{1}H NMR (400 MHz, $CDCl_3$) δ 7.80 (d, *J* = 7.6 Hz, 2H), 7.27 (d, *J* = 8.4 Hz, 2H), 7.13–7.10 (m, 2H), 7.00–6.92 (m, 2H), 4.82 (s, 1H), 4.07 (d, *J* = 6.0 Hz, 2H), 2.36 (s, 3H); ^{13}C NMR (100 MHz, $CDCl_3$) δ 162.5 (d, $^1J_{C,F}$ = 248 Hz), 143.7, 136.8, 133.5, 133.4, 129.6, 127.5, 118.1, 115.4 (d, $^2J_{C,F}$ = 22 Hz), 83.6, 83.0, 33.6, 21.4. ^{19}F NMR (376 MHz, $CDCl_3$) δ 110.33. EI-MS (*m*/*z*, relative intensity): 303 (M^+, 0.78), 148 (100). HRMS (EI) calcd. for

$[C_{16}H_{14}NO_2SF]^+$: 303.0729, found 303.0732.

(5) Methyl 4-[3-(4-methylphenylsulfonamido)prop-1-ynyl]benzoate (2e)

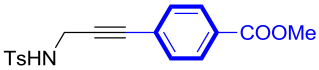

2e

The reaction of propargyl tosylamide **1e** and 4-(methoxycarbonyl)-phenylboronic acid was carried out at 50 °C for 12 h to afford **2e** (hexanes/EtOAc = 5/1) in 66% yield as a white solid. ^{1}H NMR (400 MHz, $CDCl_3$) δ 7.90 (d, *J* = 7.6 Hz, 2H), 7.81 (d, *J* = 7.2 Hz, 2H), 7.27 (d, *J* = 7.6 Hz, 2H), 7.16 (d, *J* = 7.6 Hz, 2H), 4.87 (t, *J* = 5.6 Hz, 1H), 4.10 (d, *J* = 5.6 Hz, 2H), 3.92 (s, 3 H), 2.36 (s, 3 H); ^{13}C NMR (100 MHz, $CDCl_3$) δ 166.4, 143.7, 136.8, 131.4, 129.6, 129.2, 127.5, 126.7, 86.3, 83.8, 52.3, 33.6, 21.4. EI-MS (*m*/*z*, relative intensity): 343 (M^+, 1), 188 (100). HRMS (EI) calcd. for $[C_{18}H_{17}NO_4S]^+$: 343.0878, found 343.0879.

(6) *N*-[3-(4-Methoxyphenyl)-prop-2-ynyl]-*p*-toluenesulfonamide (2f)

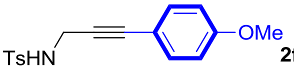

2f

The product could not be separated from the unreacted starting material. Based on the ^{1}H NMR of the mixture, **2f** was produced in 31% yield.

(7) *tert*-Butyl 4-phenylbut-3-ynyl(tosyl)carbamate (3)

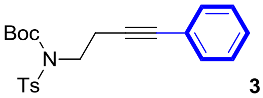

3

The reaction was carried out at 50 °C for 12 h to give **3** (hexanes/EtOAc = 5/1) in 48% yield as a viscous oil. ^{1}H NMR (400 MHz, $CDCl_3$) δ 7.83 (d, *J* = 7.6 Hz,

2H), 7.37 (s, 2H), 7.27 (s, 5H), 4.09 (t, J = 7.2 Hz, 1H), 2.88 (t, J = 7.2 Hz, 2H), 2.42 (s, 3H), 1.34 (s, 9 H); ^{13}C NMR (100 MHz, $CDCl_3$) δ 150.8, 144.2, 137.2, 131.6, 129.2, 128.1, 127.9, 127.8, 123.4, 86.0, 84.5, 82.5, 45.3, 27.8, 21.5, 20.8. EI-MS (*m*/*z*, relative intensity): 399 (M^+, 0.46), 128 (100). HRMS (EI) calcd. for $[C_{22}H_{25}NO_4S]^+$: 399.1504, found 399.1471.

(8) *tert*-Butyl 5-phenylpent-4-ynyl(tosyl)carbamate (4)

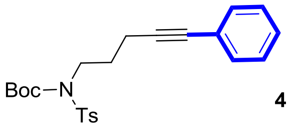

4

The reaction was run at 50 °C for 12 h to give **4** (hexanes/EtOAc = 5/1) in 68% yield as a viscous oil. ^{1}H NMR (400 MHz, $CDCl_3$) δ 7.81 (d, J = 7.6 Hz, 2H), 7.41–7.40 (m, 2H), 7.31–7.27 (m, 5H), 3.98 (t, J = 7.2 Hz, 1H), 2.50 (t, J = 6.8 Hz, 2H), 2.43 (s, 3H), 2.07 (t, J = 6.8 Hz, 1H), 1.34 (s, 9H); ^{13}C NMR (100 MHz, $CDCl_3$) δ 150.9, 144.1, 137.3, 131.6, 129.2, 128.1, 127.8, 127.6, 123.7, 88.7, 84.2, 81.2, 46.4, 29.2, 27.8, 21.6, 17.0. EI-MS (*m*/*z*, relative intensity): 313 (M^+−100, 0.19), 57 (100). HRMS (EI) calcd. for $[C_{18}H_{19}NO_2S]^+$: 313.1137, found 313.1139.

(9) 4-Phenylbut-3-yn-1-yltosylate (5)

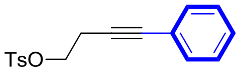

5

The reaction was run at room temperature for 12 h to give **6** (hexanes/EtOAc = 5/1) in 71% yield as a pale yellow oil. Its spectroscopic data were in accord

with those reported in the literature [4]. ^{1}H NMR (400 MHz, $CDCl_3$) δ 7.81 (d, *J* = 7.6 Hz, 2H), 7.32–7.26 (m, 7H), 4.19 (t, *J* = 6.8 Hz, 1H), 2.79 (t, *J* = 6.8 Hz, 2H), 2.42 (s, 3H); ^{13}C NMR (100 MHz, $CDCl_3$) δ 145.0, 132.9, 131.7, 130.0, 128.3, 128.2, 128.0, 123.0, 83.9, 82.7, 67.8, 21.7, 20.4.

(10) 4-Methyl-*N*,*N*-bis(3-phenylprop-2-ynyl)benzenesulfonamide (6)

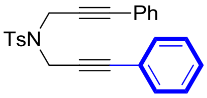

6

The reaction was run at room temperature for 12 h to give **6** (hexanes/EtOAc = 5/1) in 71% yield as a pale yellow oil. Its spectroscopic data were in accord with those reported in the literature [5]. ^{1}H NMR (400 MHz, $CDCl_3$) δ 7.79 (d, *J* = 7.6 Hz, 2H), 7.31–7.20 (m, 12H), 7.27 (s, 5H), 4.45 (s, 4H), 2.31 (s, 3H); ^{13}C NMR (100 MHz, $CDCl_3$) δ 143.8, 135.3, 131.7, 129.6, 128.5, 128.1, 128.0, 122.2, 85.8, 81.6, 37.5, 21.4.

(8) (3-Benzyloxyprop-1-ynyl)benzene (7)

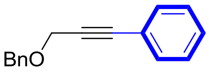

7

The reaction was run at 50 °C for 12 h to give **7** (hexanes/EtOAc = 5/1) in 71% yield as a yellow oil. Its spectroscopic data were in accord with those reported in the literature [6]. ^{1}H NMR (400 MHz, $CDCl_3$) δ 7.48–7.46 (m, 2H), 7.41–7.30 (m, 8H), 4.69 (s, 2H), 4.41 (s, 2H); ^{13}C NMR (100 MHz, $CDCl_3$) δ 137.4, 131.8, 128.4, 128.3, 128.1, 127.9, 122.6, 86.5, 85.0, 71.6, 57.9.

(11) *N*-Allyl-4-methyl-*N*-(3-phenylprop-2-ynyl)benzenesulfonamide (8)

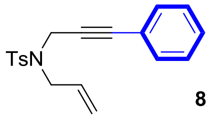

8

The reaction was run at room temperature for 12 h to give **8** (hexanes/EtOAc = 5/1) in 82% yield as a white solid. Its spectroscopic data were in accord with those reported in the literature [7]. ^{1}H NMR (400 MHz, $CDCl_3$) δ 7.77 (d, *J* = 7.6 Hz, 2H), 7.30–7.22 (m, 5H), 7.05 (d, *J* = 7.2 Hz, 2H), 5.87–5.73 (m, 1H), 5.35–5.27 (m, 2H), 4.31 (s, 2H), 3.88 (d, *J* = 6.4 Hz, 2H), 2.34 (s, 3H); ^{13}C NMR (100 MHz, $CDCl_3$) δ 143.5, 135.8, 132.0, 131.4, 129.5, 128.4, 128.1, 127.8, 122.1, 120.0, 85.7, 81.6, 49.2, 36.7, 21.4.

4. References

1. Nieto-Oberhuber, C.; Pérez-Galán, P.; Herrero-Gómez, E.; Lauterbach, T.; Rodríguez, C.; López, S.; Bour, C.; Rosellón, A.; Cárdenas, D. J.; Echavarren, A. M. *J. Am. Chem. Soc.* **2008,** *130,* 269. doi:10.1021/ja075794x
2. Lee, S. I.; Park, S. Y.; Park, J. H.; Jung, I. G.; Choi, S. Y.; Chung, Y. K. *J. Org. Chem.* **2006,** *71,* 91. doi:10.1021/jo051685u
3. Shintani, R.; Nakatsu, H.; Takatsu, K.; Hayashi, T. *Chem.–Eur. J.* **2009,** *15,* 8692. doi:10.1002/chem.200901463
4. Collins, C. J.; Hanack, M.; Stutz, H.; Auchter, G.; Schoberth, W. *J. Org. Chem.* **1983,** *48,* 5260. doi:10.1021/jo00174a021
5. Lian, J.-J.; Chen, P.-C.; Lin, Y.-P.; Ting, H.-C.; Liu, R. *J. Am. Chem. Soc.* **2006,** *128,* 11372. doi:10.1021/ja0643826
6. Bolte, B.; Odabachian, Y.; Gagosz, F. *J. Am. Chem. Soc.* **2010,** *132,* 7294. doi:10.1021/ja1020469

7. Chen, M.; Weng, Y.; Guo, M.; Zhang, H.; Lei, A. *Angew. Chem., Int. Ed.* **2008,** *47,* 2279. doi:10.1002/anie.200704452

5. ^{1}H and ^{13}C NMR spectra

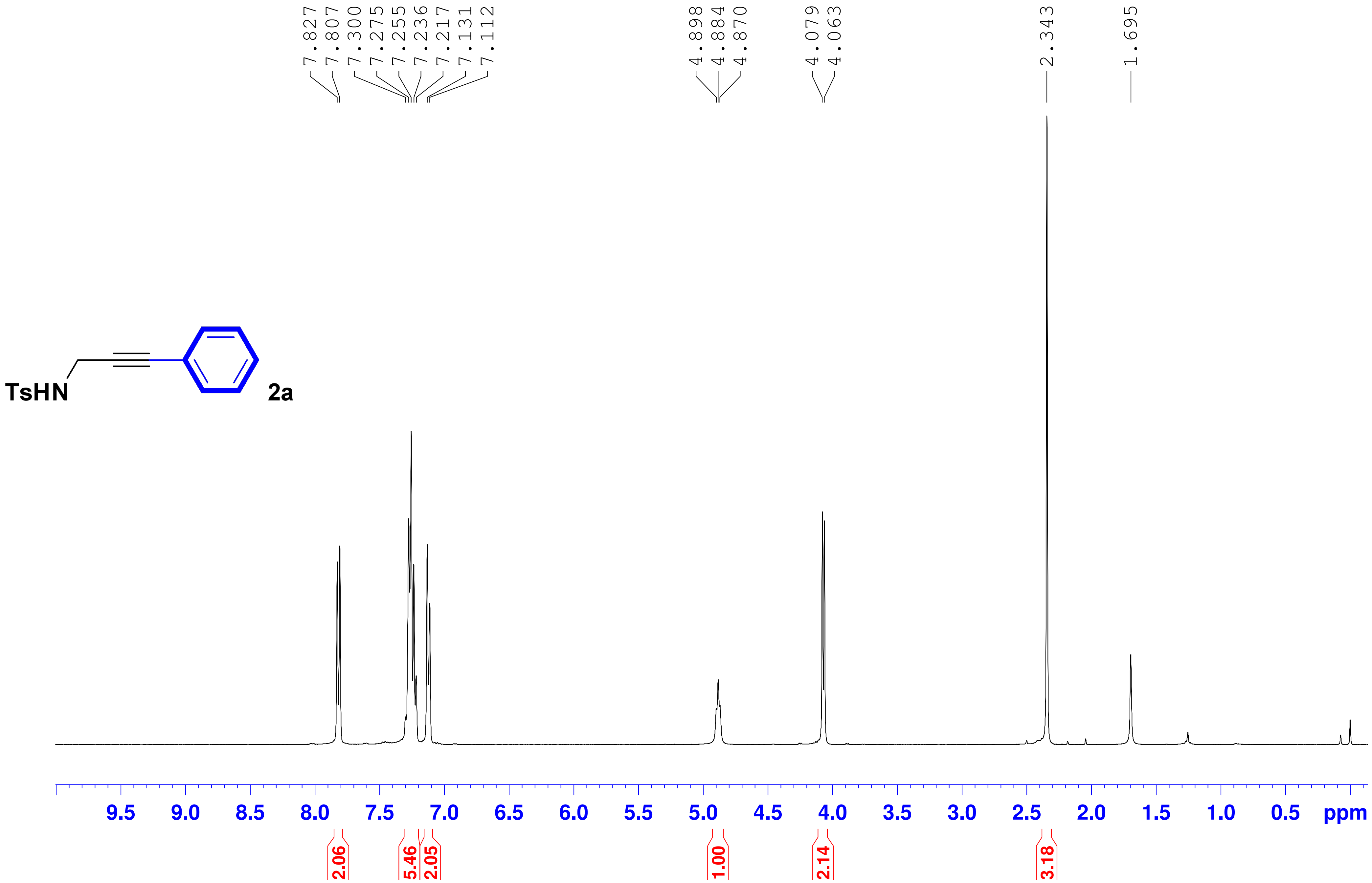

7.827
7.807
7.300
7.275
7.255
7.236
7.217
7.131
7.112
4.898
4.884
4.870
4.079
4.063
2.343
1.695
TsHN
2a
9.5
9.0
8.5
8.0
7.5
7.0
6.5
6.0
5.5
5.0
4.5
4.0
3.5
3.0
2.5
2.0
1.5
1.0
0.5
ppm
2.06
5.46
2.05
1.00
2.14
3.18

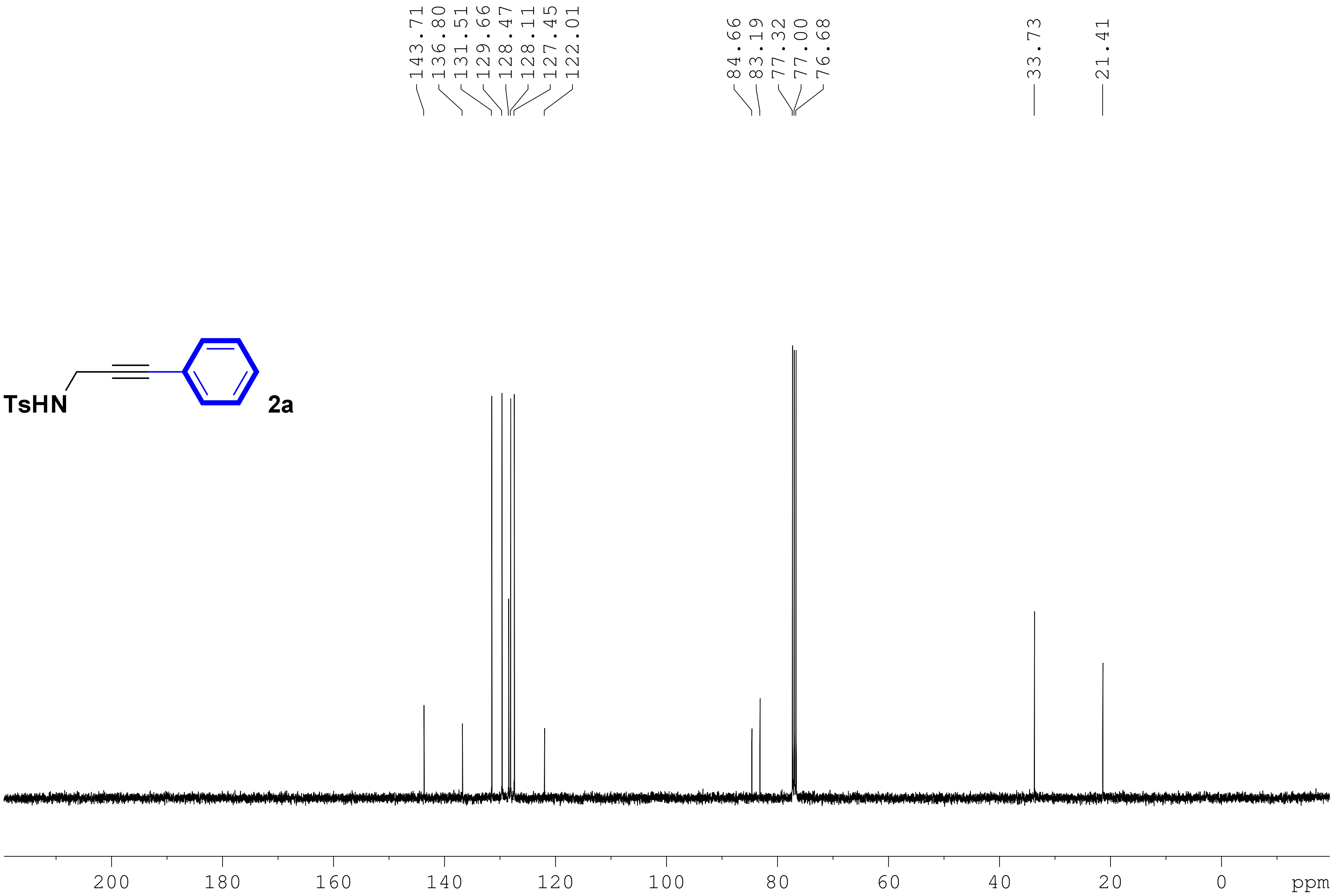

143.71
136.80
131.51
129.66
128.47
128.11
127.45
122.01
84.66
83.19
77.32
77.00
76.68
33.73
21.41
TsHN
2a
200
180
160
140
120
100
80
60
40
20
0
ppm

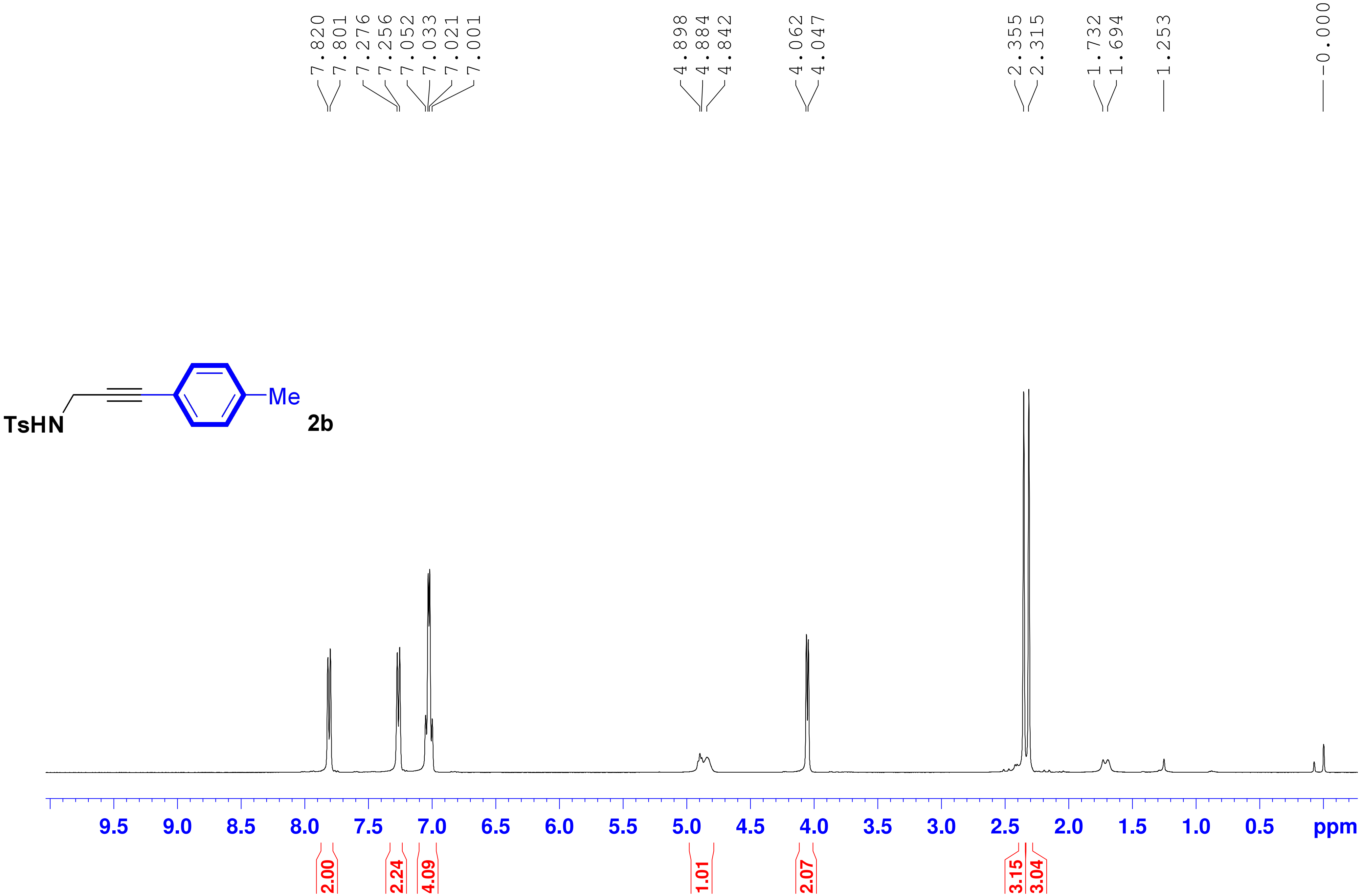
7.820
7.801
7.276
7.256
7.052
7.033
7.021
7.001
4.898
4.884
4.842
4.062
4.047
2.355
2.315
1.732
1.694
1.253
-0.000
TsHN
Me
2b
9.5 9.0 8.5 8.0 7.5 7.0 6.5 6.0 5.5 5.0 4.5 4.0 3.5 3.0 2.5 2.0 1.5 1.0 0.5 ppm
2.00
2.24
4.09
1.01
2.07
3.15
3.04

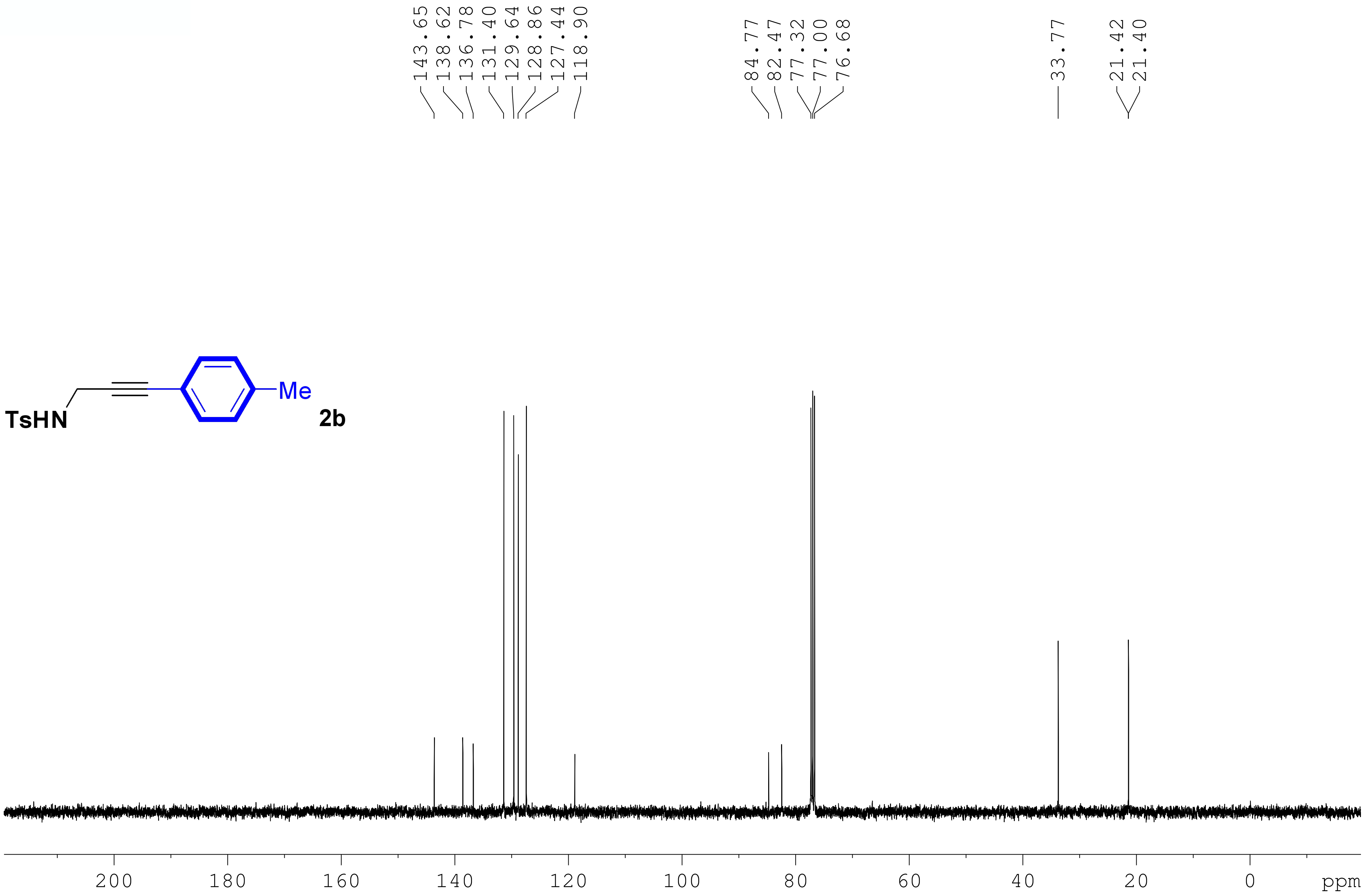

143.65
138.62
136.78
131.40
129.64
128.86
127.44
118.90
84.77
82.47
77.32
77.00
76.68
33.77
21.42
21.40
TsHN
Me
200
180
160
140
120
100
80
60
40
20
0
ppm

2b

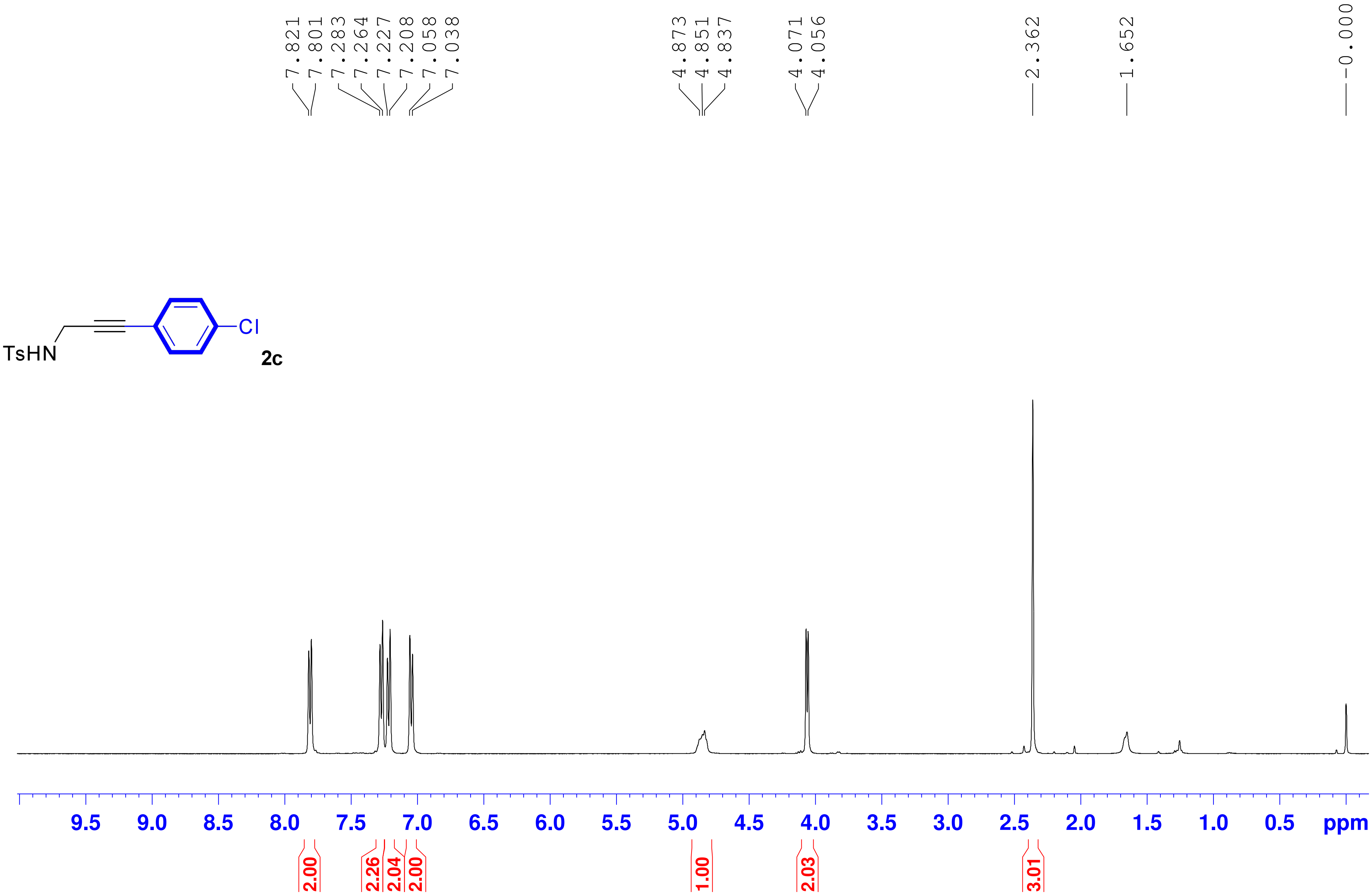

7.821
7.801
7.283
7.264
7.227
7.208
7.058
7.038
4.873
4.851
4.837
4.071
4.056
2.362
1.652
-0.000
TsHN
Cl
2c
9.5 9.0 8.5 8.0 7.5 7.0 6.5 6.0 5.5 5.0 4.5 4.0 3.5 3.0 2.5 2.0 1.5 1.0 0.5 ppm
2.00
2.26
2.04
2.00
1.00
2.03
3.01

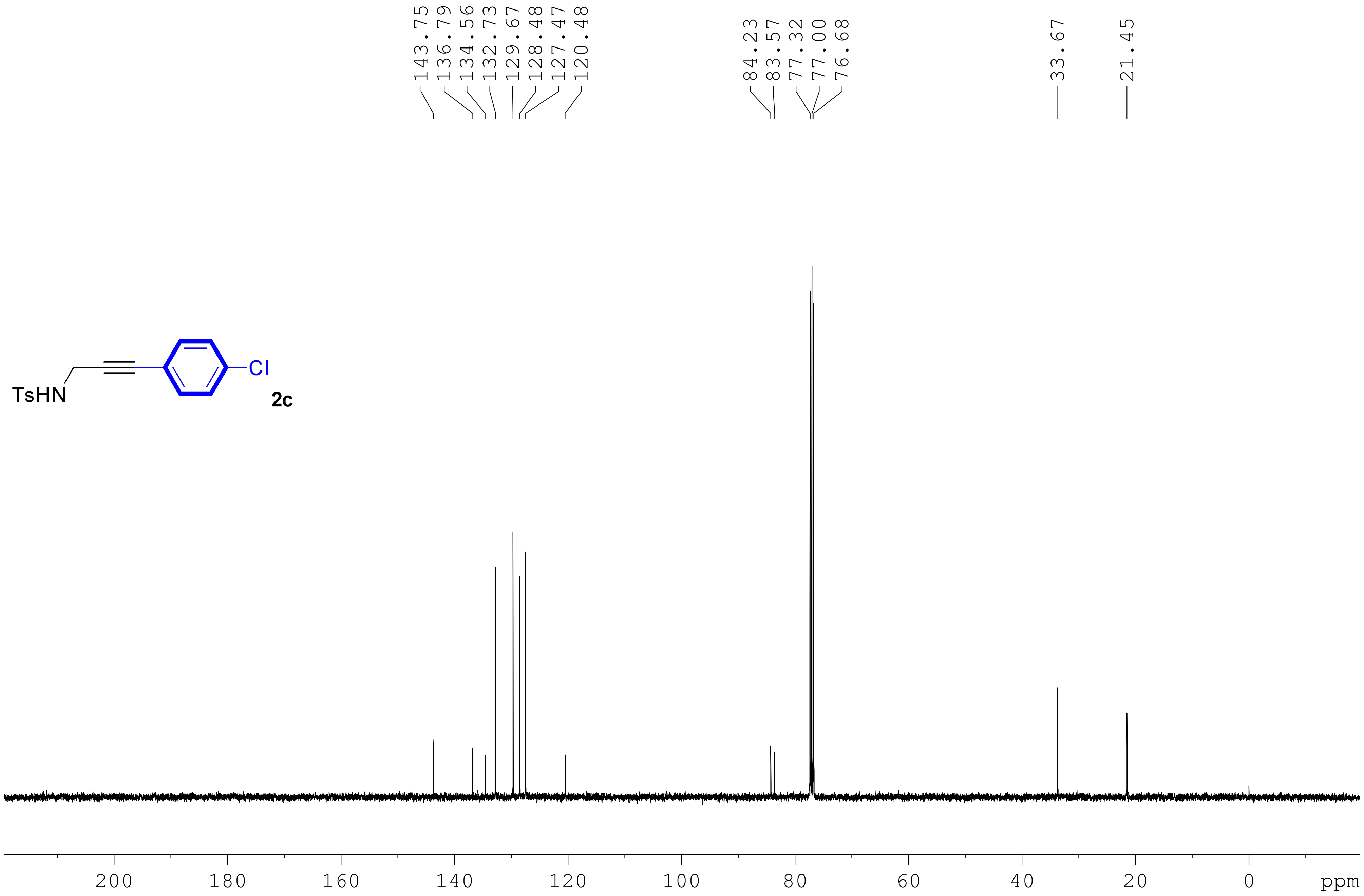

143.75
136.79
134.56
132.73
129.67
128.48
127.47
120.48
84.23
83.57
77.32
77.00
76.68
33.67
21.45
TsHN
Cl
2c
200
180
160
140
120
100
80
60
40
20
0
ppm

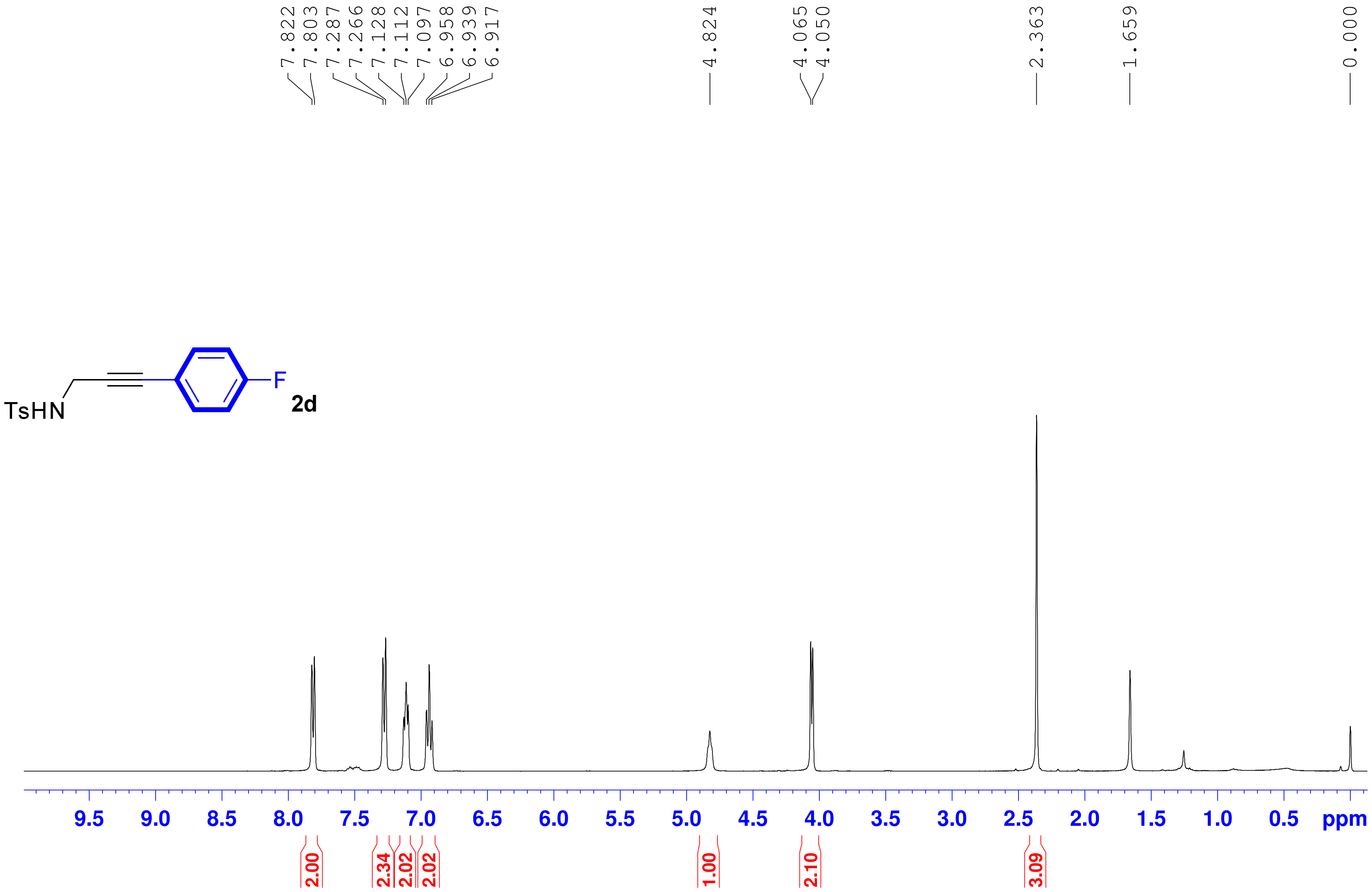

TsHN
F
7.822
7.803
7.287
7.266
7.128
7.112
7.097
6.958
6.939
6.917
4.824
4.065
4.050
2.363
1.659
0.000
9.5
9.0
8.5
8.0
7.5
7.0
6.5
6.0
5.5
5.0
4.5
4.0
3.5
3.0
2.5
2.0
1.5
1.0
0.5
ppm
2.00
2.34
2.02
2.02
1.00
2.10
3.09

2d

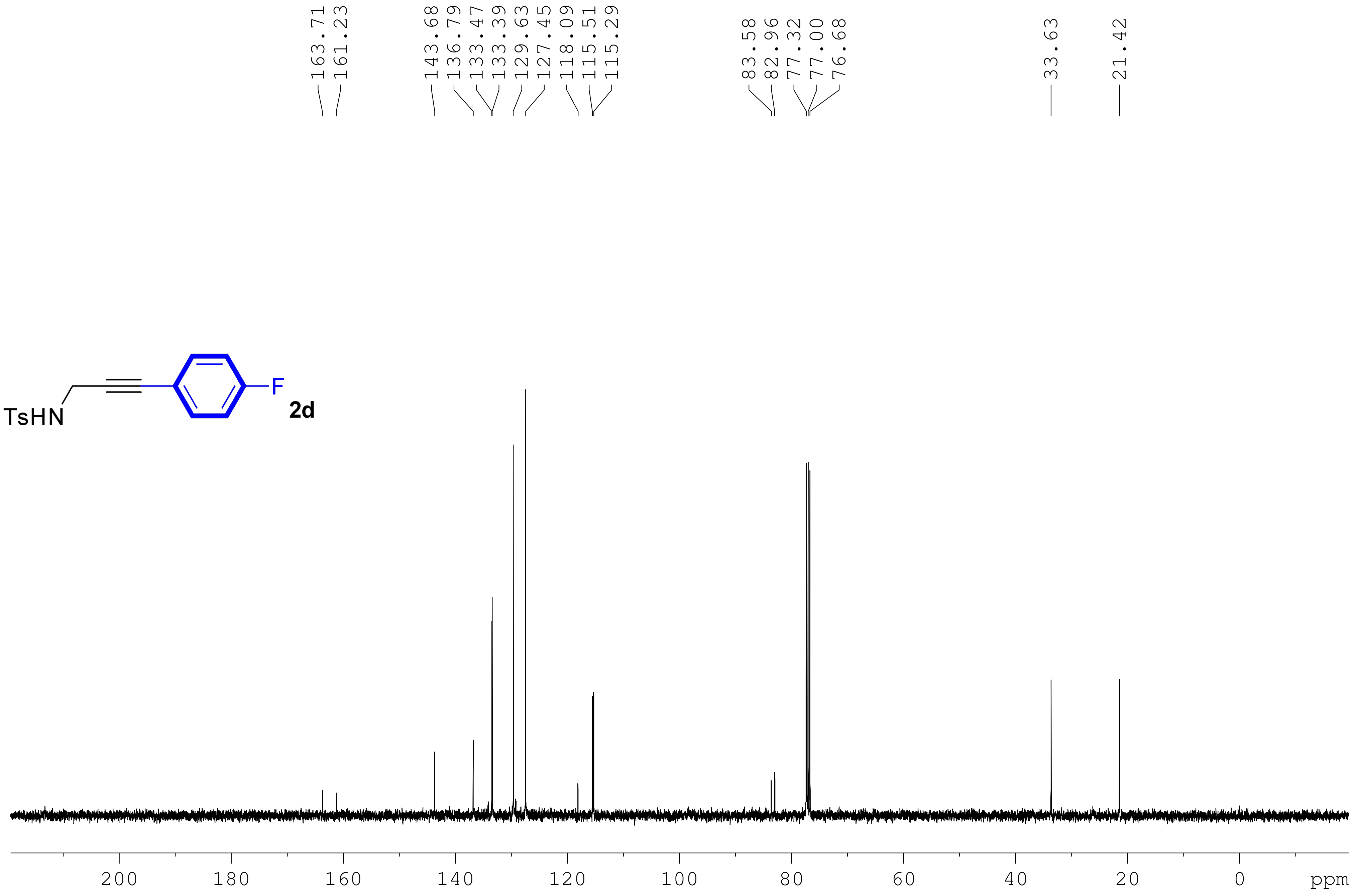

163.71
161.23
143.68
136.79
133.47
133.39
129.63
127.45
118.09
115.51
115.29
83.58
82.96
77.32
77.00
76.68
33.63
21.42
TsHN
F
2d
200
180
160
140
120
100
80
60
40
20
0
ppm

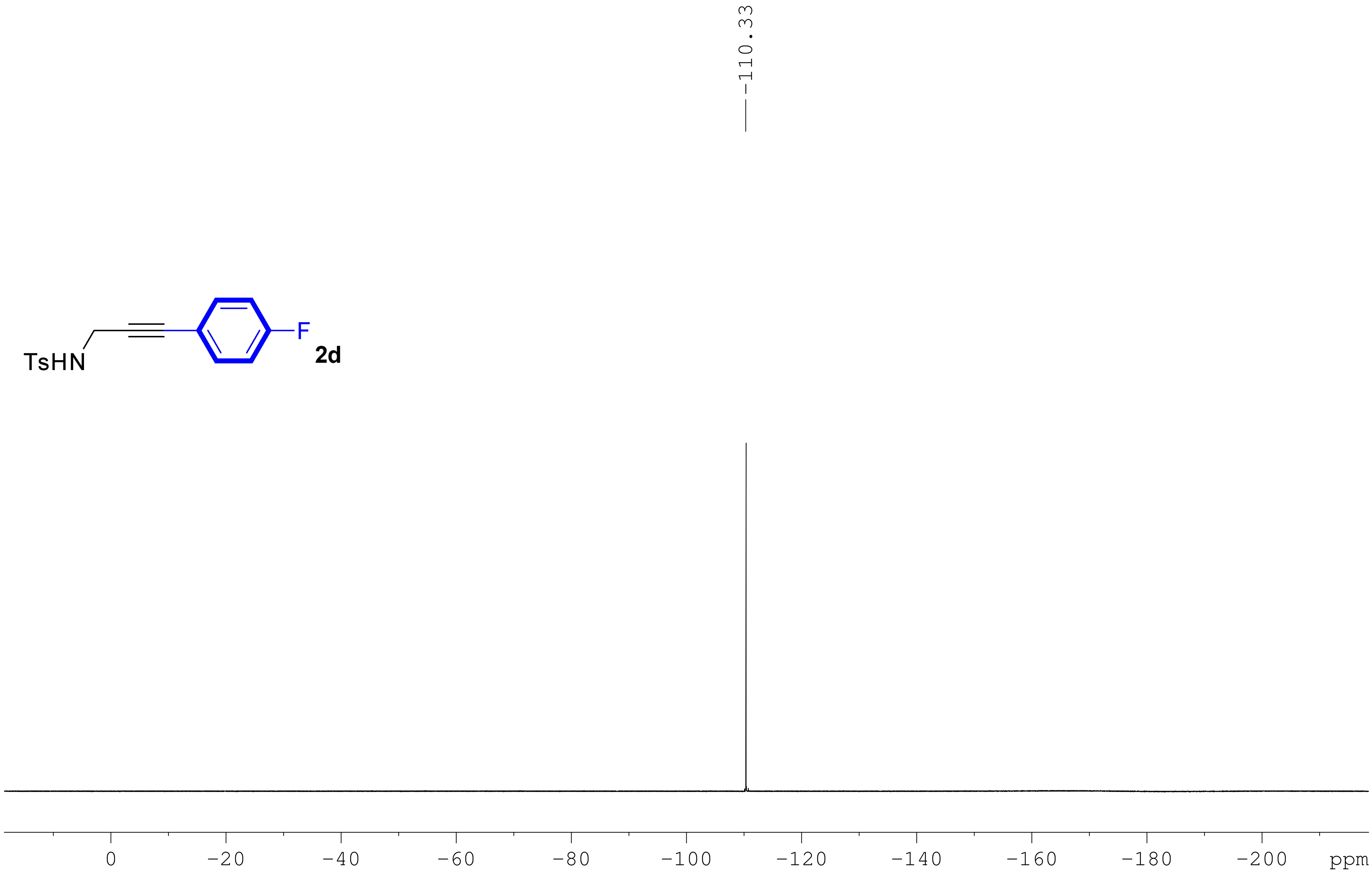
-110.33
TsHN
F
2d
0
-20
-40
-60
-80
-100
-120
-140
-160
-180
-200
ppm

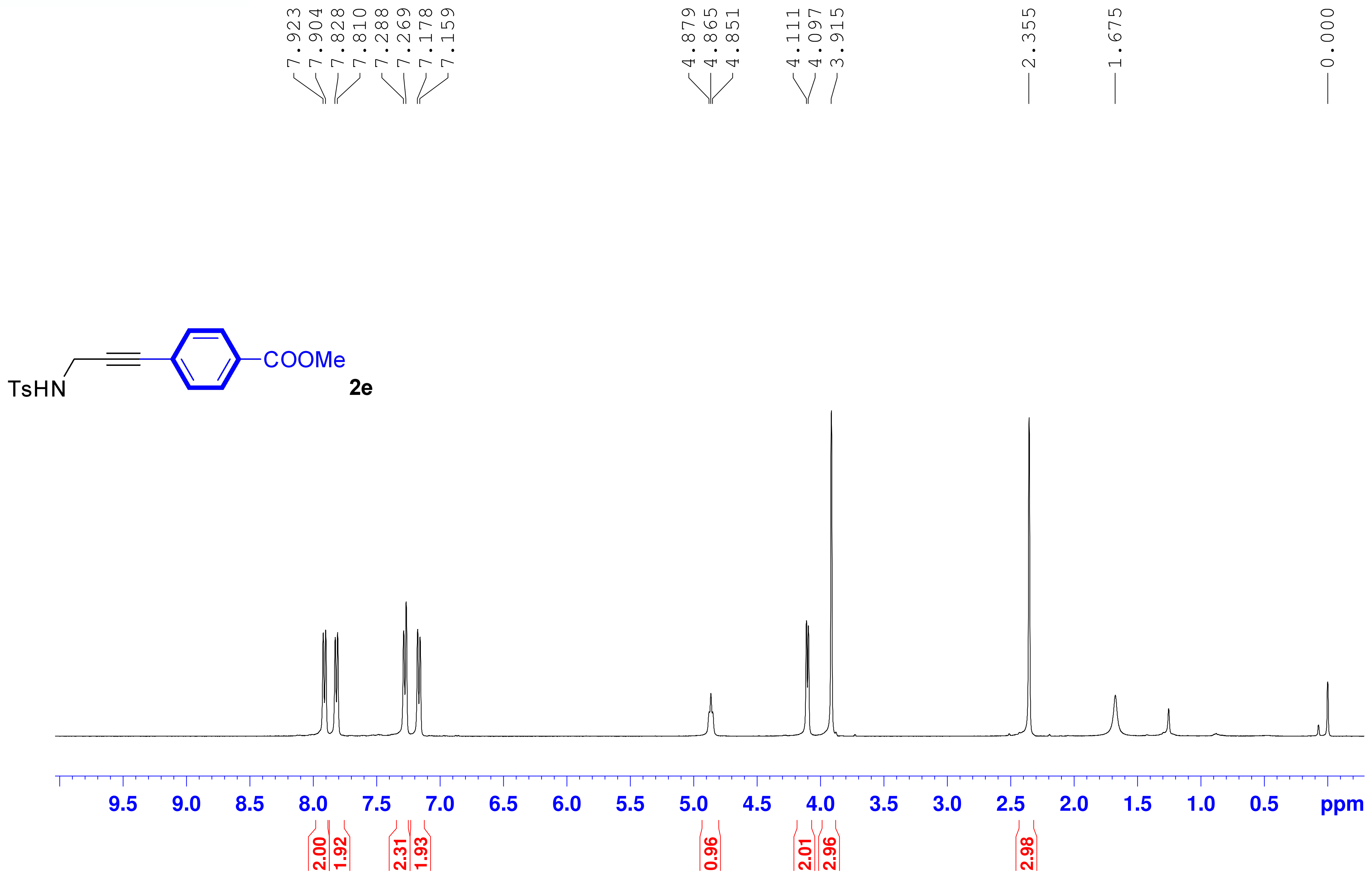
7.923
7.904
7.828
7.810
7.288
7.269
7.178
7.159
4.879
4.865
4.851
4.111
4.097
3.915
2.355
1.675
0.000
TsHN
COOMe
2e
9.5
9.0
8.5
8.0
7.5
7.0
6.5
6.0
5.5
5.0
4.5
4.0
3.5
3.0
2.5
2.0
1.5
1.0
0.5
ppm
2.00
1.92
2.31
1.93
0.96
2.01
2.96
2.98

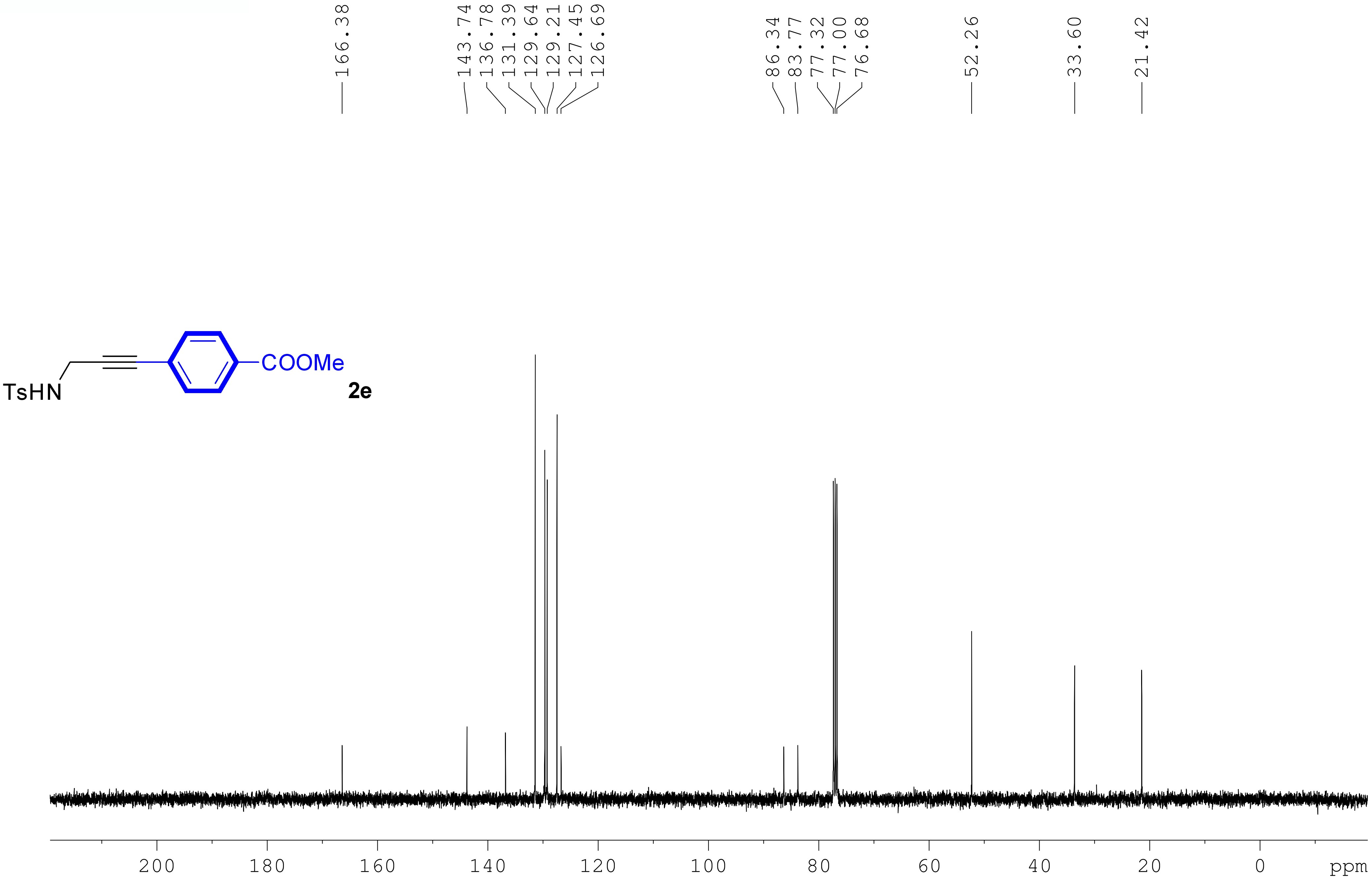

166.38
143.74
136.78
131.39
129.64
129.21
127.45
126.69
86.34
83.77
77.32
77.00
76.68
52.26
33.60
21.42
TsHN
COOMe
2e
200
180
160
140
120
100
80
60
40
20
0
ppm

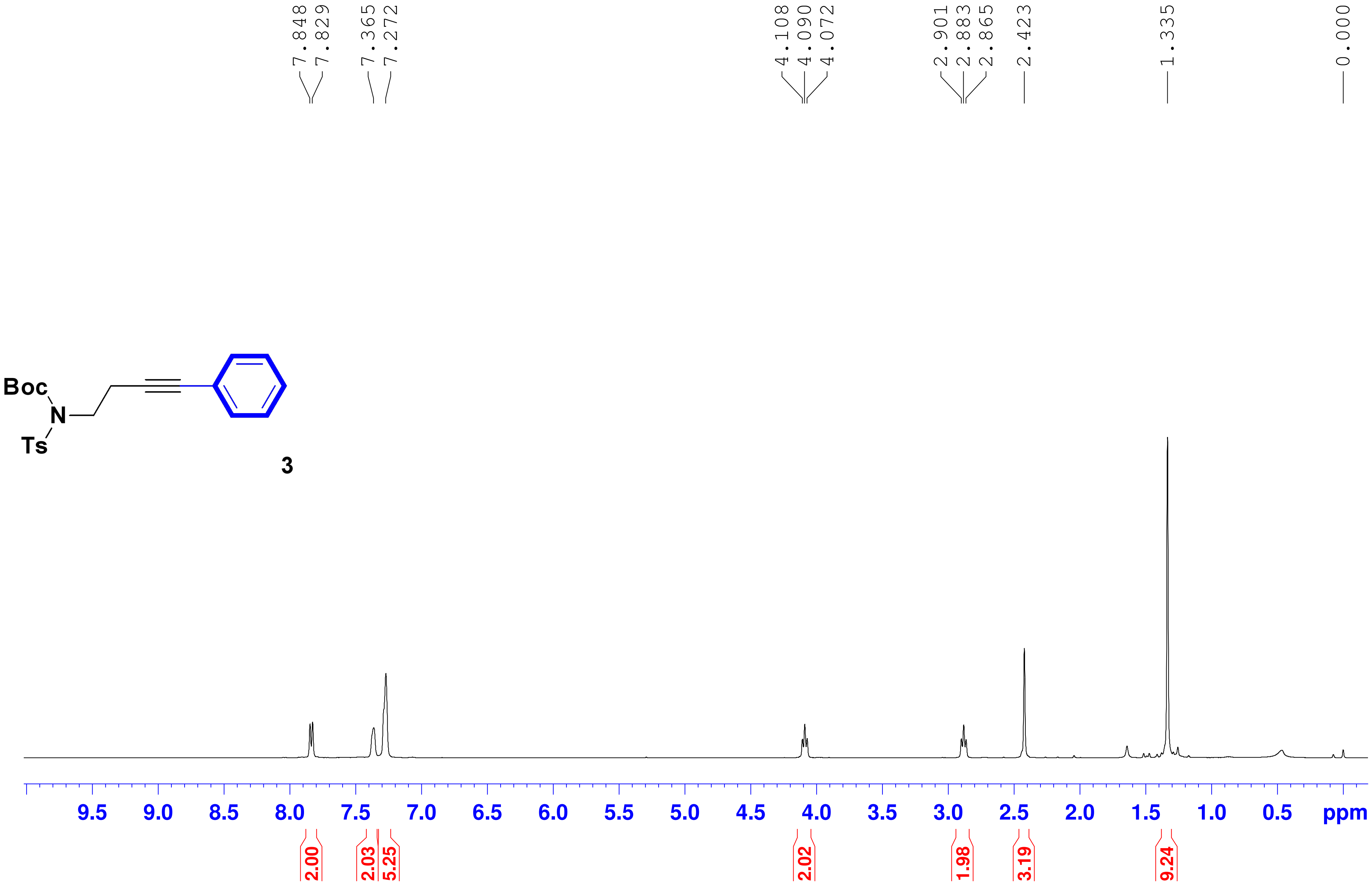
7.848
7.829
7.365
7.272
4.108
4.090
4.072
2.901
2.883
2.865
2.423
1.335
0.000
Boc
N
Ts
3
9.5
9.0
8.5
8.0
7.5
7.0
6.5
6.0
5.5
5.0
4.5
4.0
3.5
3.0
2.5
2.0
1.5
1.0
0.5
ppm
2.00
2.03
5.25
2.02
1.98
3.19
9.24

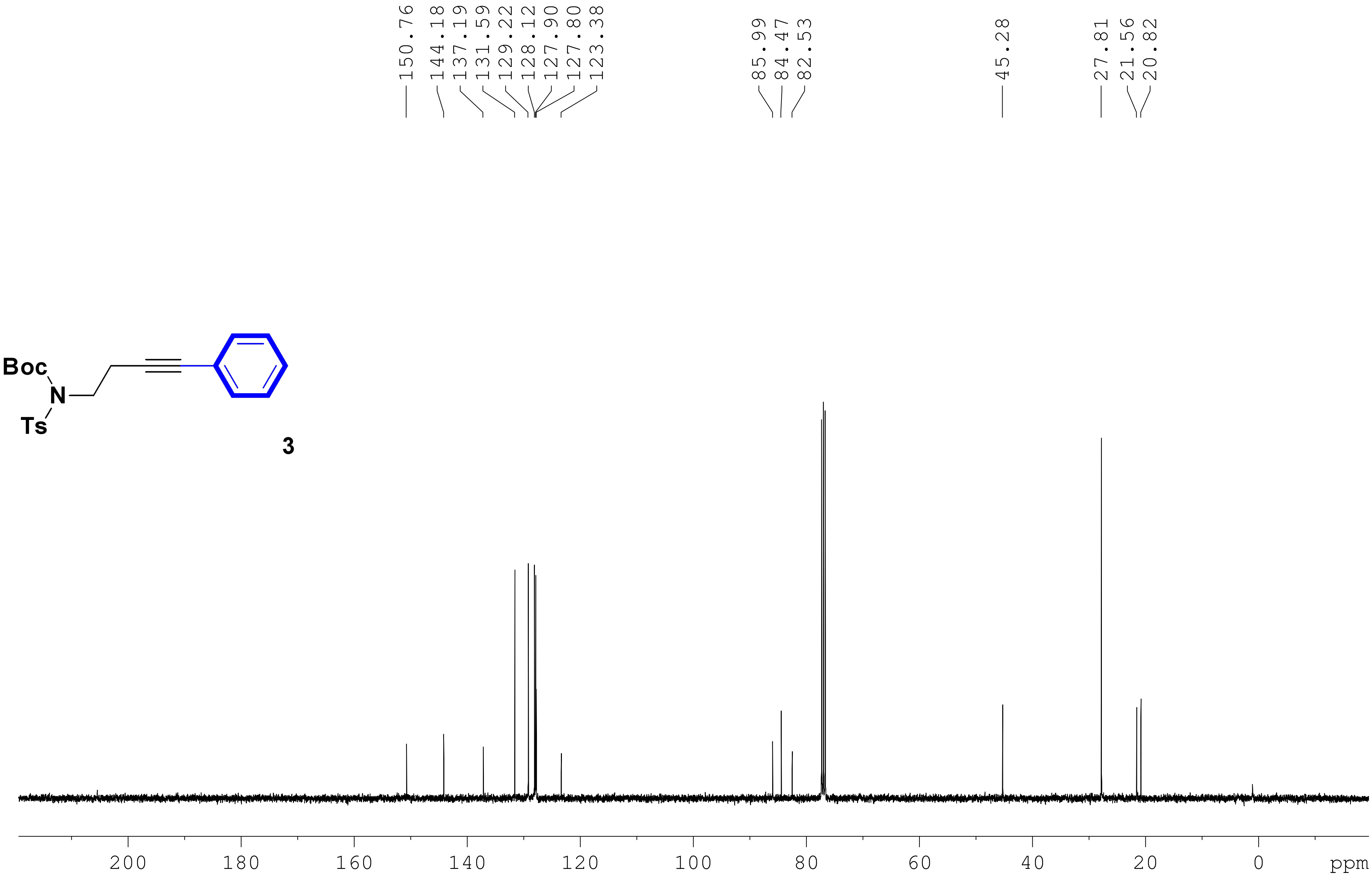
Boc
N
Ts
3
150.76
144.18
137.19
131.59
129.22
128.12
127.90
127.80
123.38
85.99
84.47
82.53
45.28
27.81
21.56
20.82
200
180
160
140
120
100
80
60
40
20
0
ppm

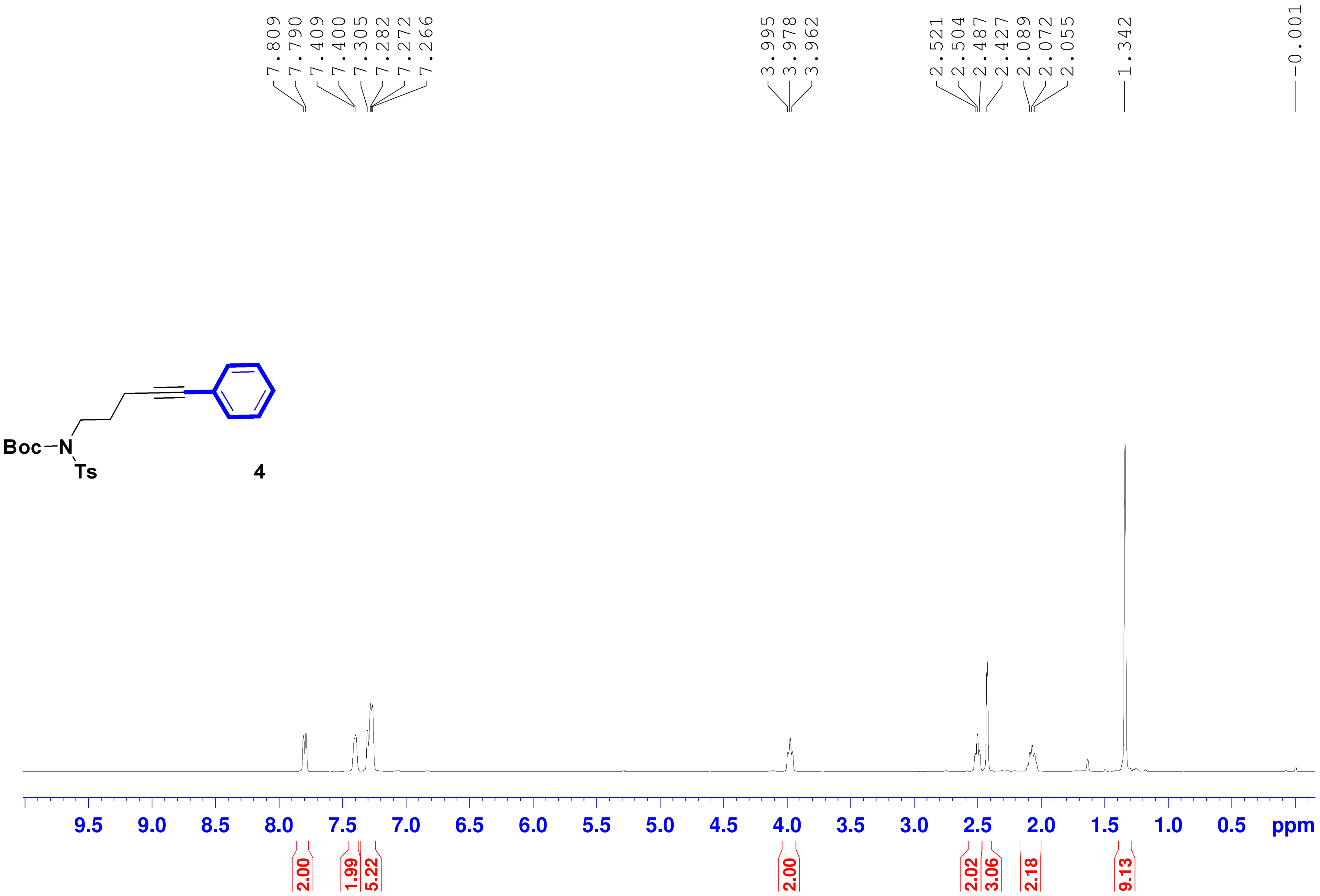
7.809
7.790
7.409
7.400
7.305
7.282
7.272
7.266
3.995
3.978
3.962
2.521
2.504
2.487
2.427
2.089
2.072
2.055
1.342
-0.001
Boc
N
Ts
4
9.5
9.0
8.5
8.0
7.5
7.0
6.5
6.0
5.5
5.0
4.5
4.0
3.5
3.0
2.5
2.0
1.5
1.0
0.5
ppm
2.00
1.99
5.22
2.00
2.02
3.06
2.18
9.13

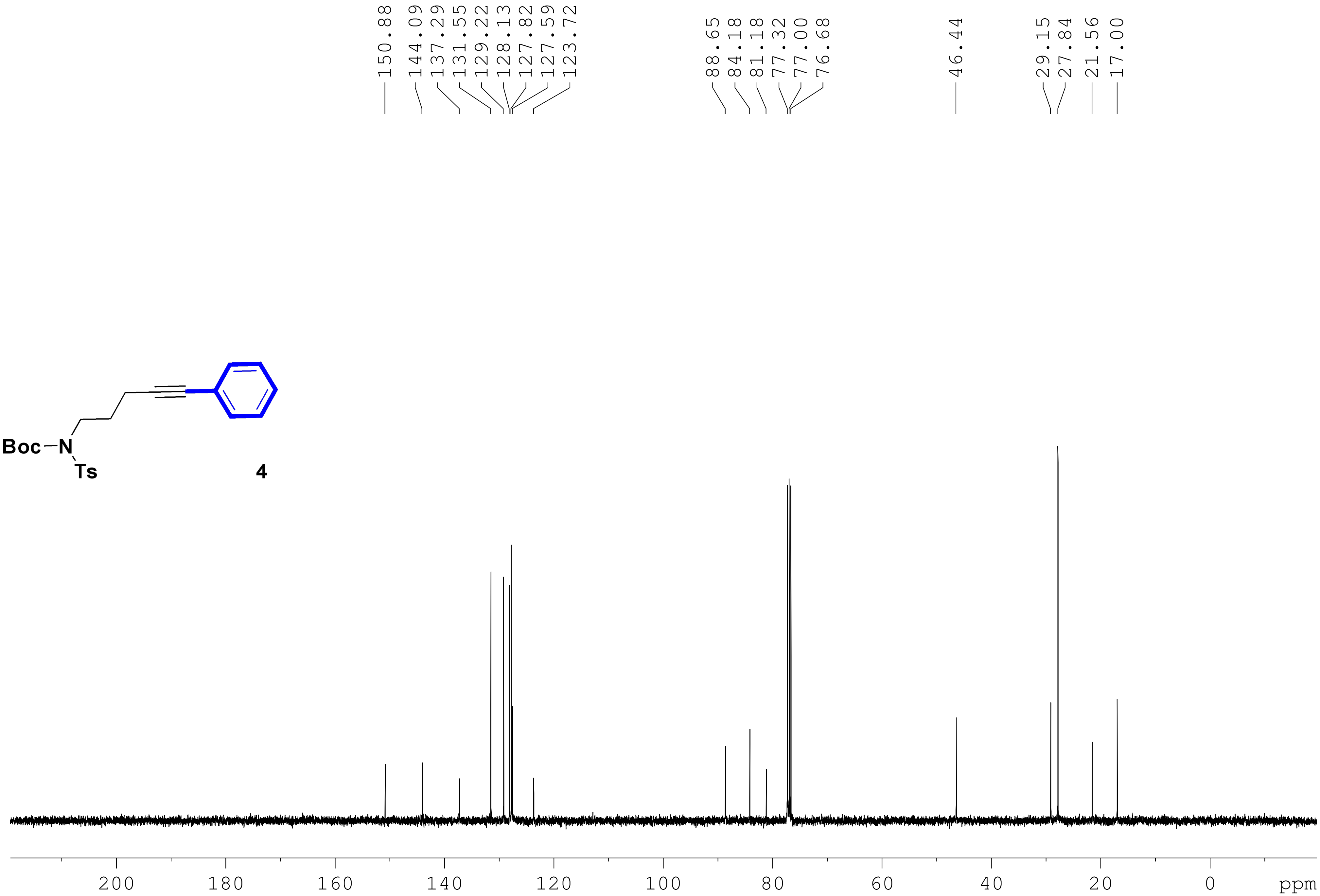
150.88
144.09
137.29
131.55
129.22
128.13
127.82
127.59
123.72
88.65
84.18
81.18
77.32
77.00
76.68
46.44
29.15
27.84
21.56
17.00
Boc
N
Ts
200
180
160
140
120
100
80
60
40
20
0
ppm

4

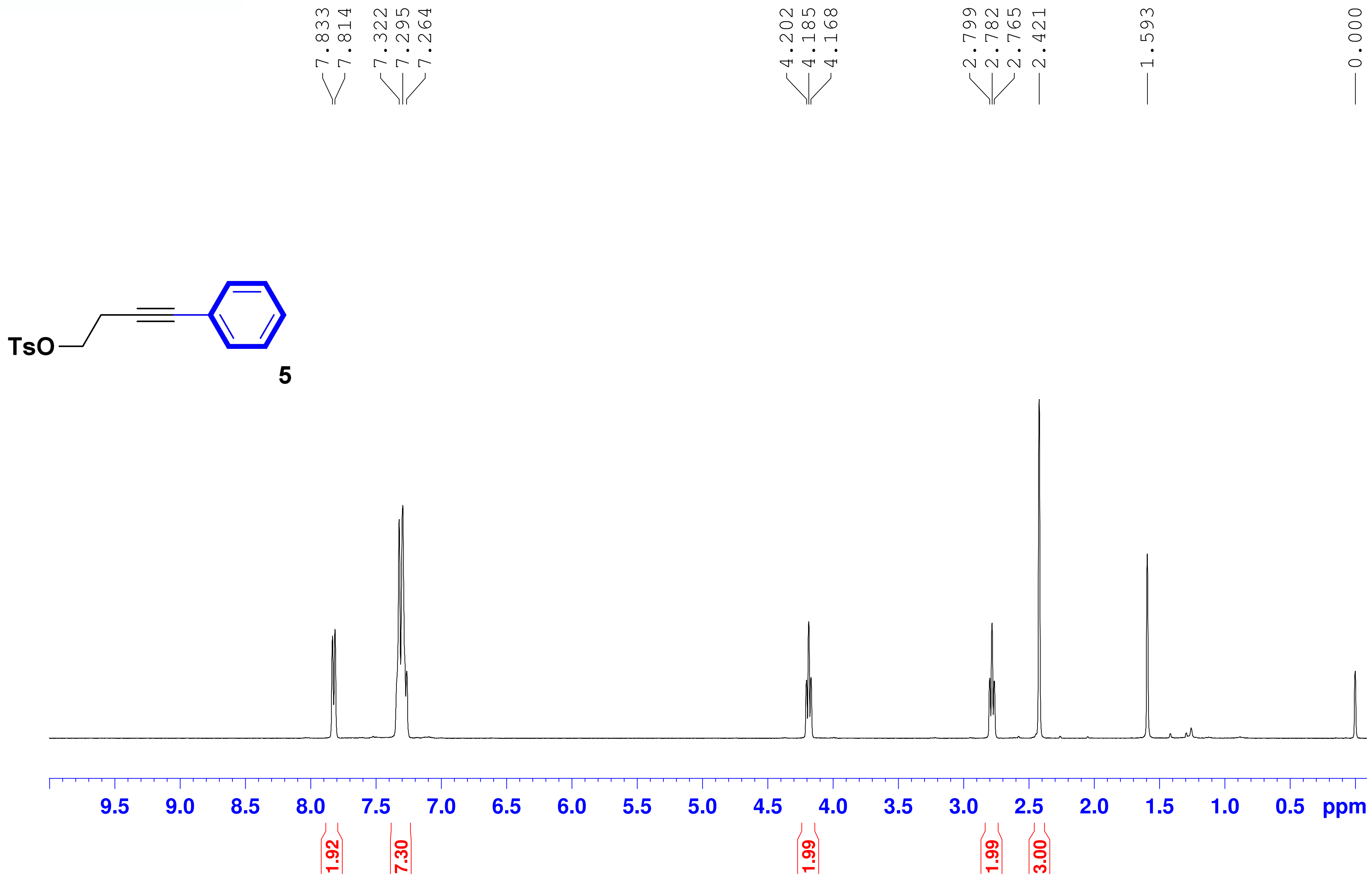
7.833
7.814
7.322
7.295
7.264
4.202
4.185
4.168
2.799
2.782
2.765
2.421
1.593
0.000
TsO
5
9.5
9.0
8.5
8.0
7.5
7.0
6.5
6.0
5.5
5.0
4.5
4.0
3.5
3.0
2.5
2.0
1.5
1.0
0.5
ppm
1.92
7.30
1.99
1.99
3.00

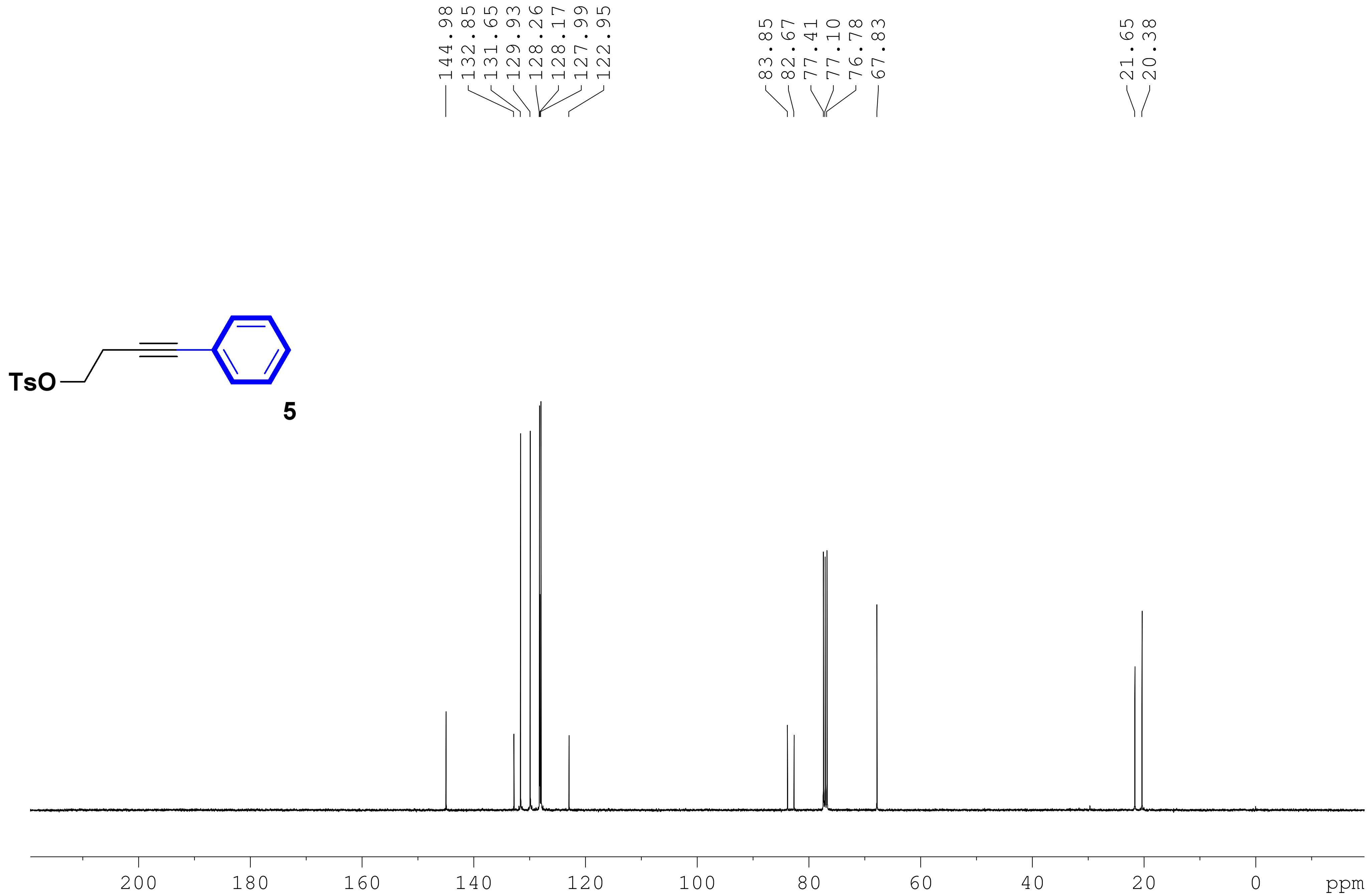
144.98
132.85
131.65
129.93
128.26
128.17
127.99
122.95
83.85
82.67
77.41
77.10
76.78
67.83
21.65
20.38
TsO
5
200
180
160
140
120
100
80
60
40
20
0
ppm

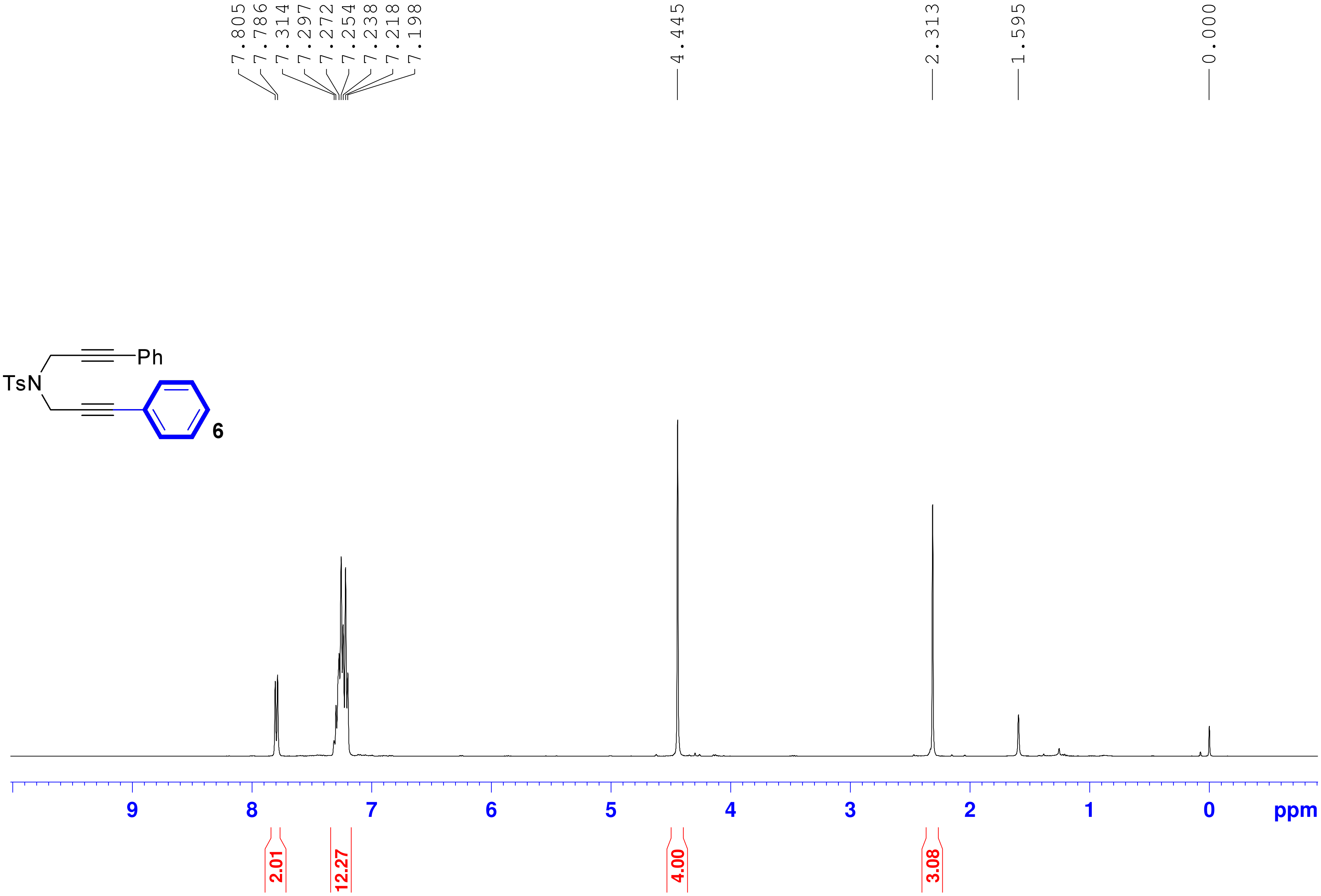
7.805
7.786
7.314
7.297
7.272
7.254
7.238
7.218
7.198
4.445
2.313
1.595
0.000
TsN
Ph
6
9
8
7
6
5
4
3
2
1
0
ppm
2.01
12.27
4.00
3.08

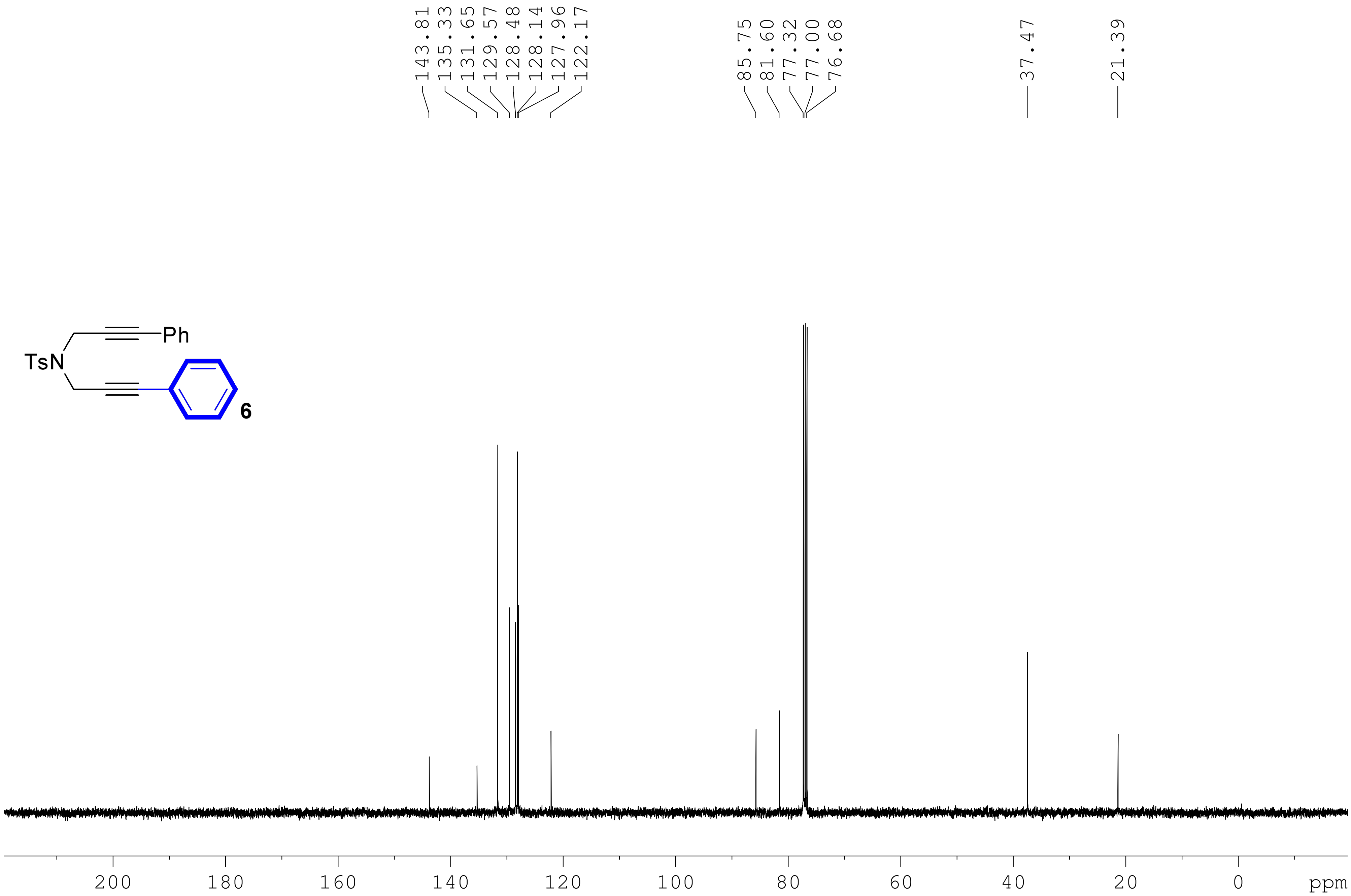
TsN
Ph
6
143.81
135.33
131.65
129.57
128.48
128.14
127.96
122.17
85.75
81.60
77.32
77.00
76.68
37.47
21.39
200
180
160
140
120
100
80
60
40
20
0
ppm

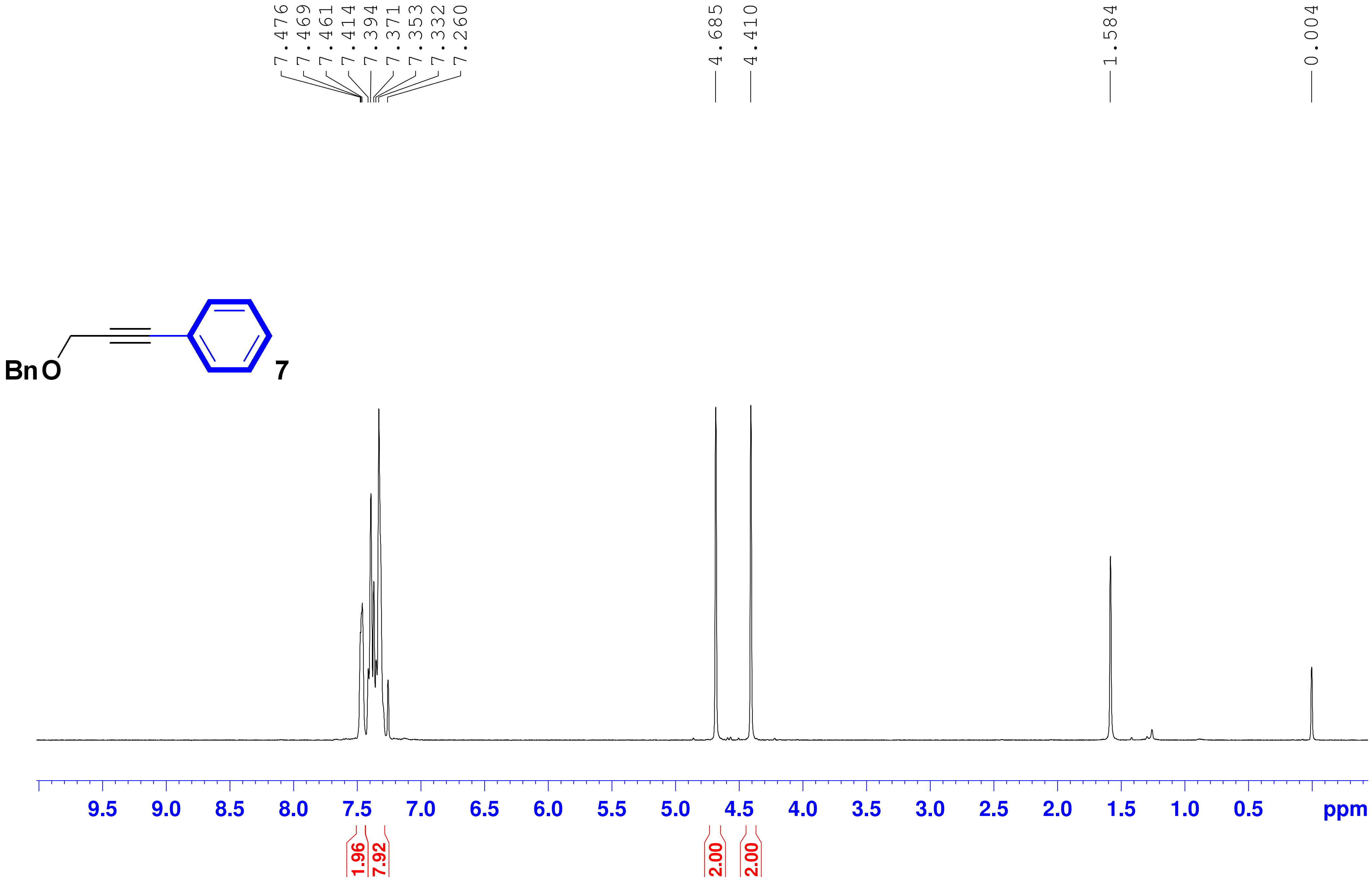
7.476
7.469
7.461
7.414
7.394
7.371
7.353
7.332
7.260
4.685
4.410
1.584
0.004
BnO
7
9.5
9.0
8.5
8.0
7.5
7.0
6.5
6.0
5.5
5.0
4.5
4.0
3.5
3.0
2.5
2.0
1.5
1.0
0.5
ppm
1.96
7.92
2.00
2.00

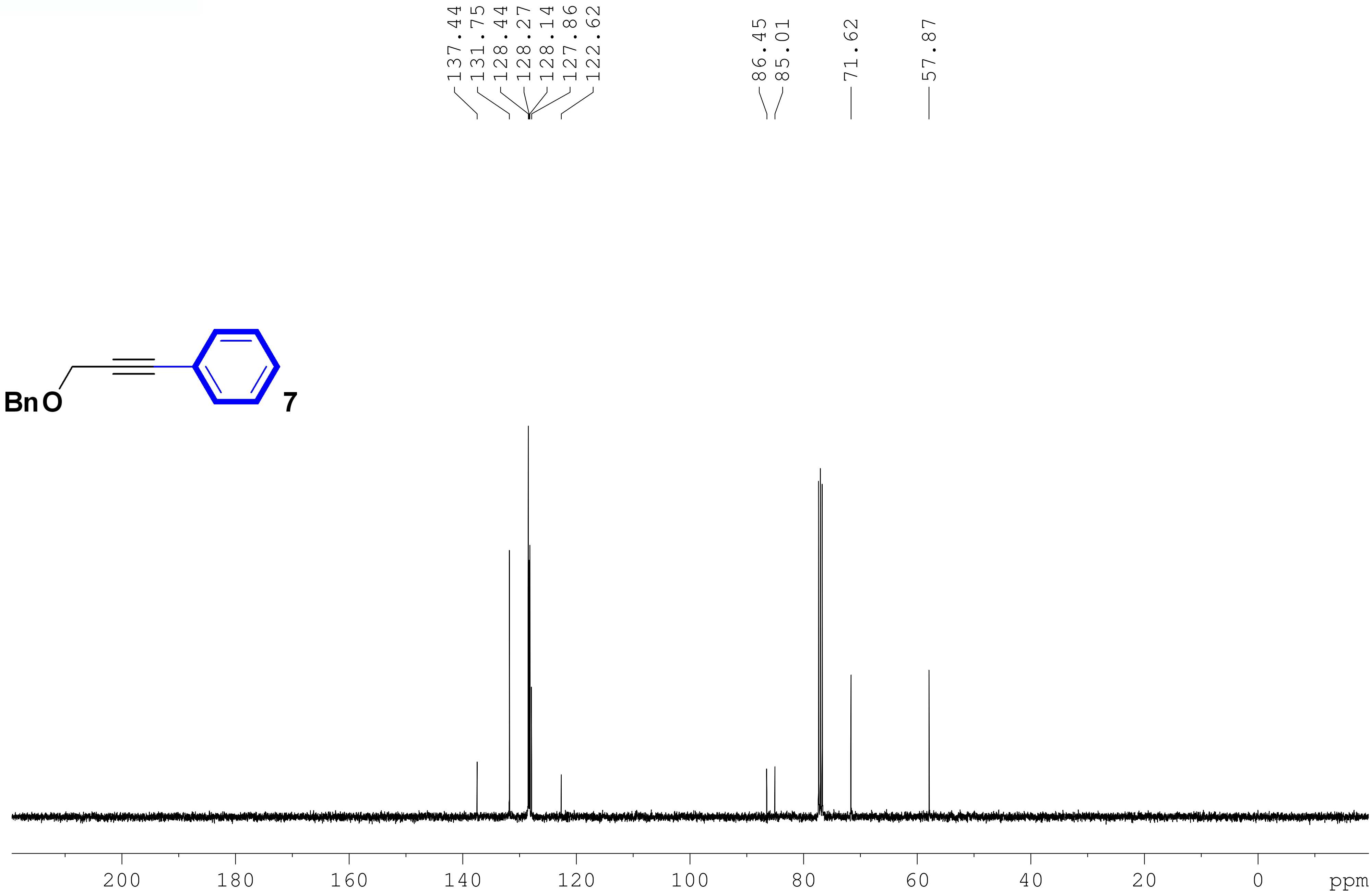
137.44
131.75
128.44
128.27
128.14
127.86
122.62
86.45
85.01
71.62
57.87
BnO
7
200
180
160
140
120
100
80
60
40
20
0
ppm

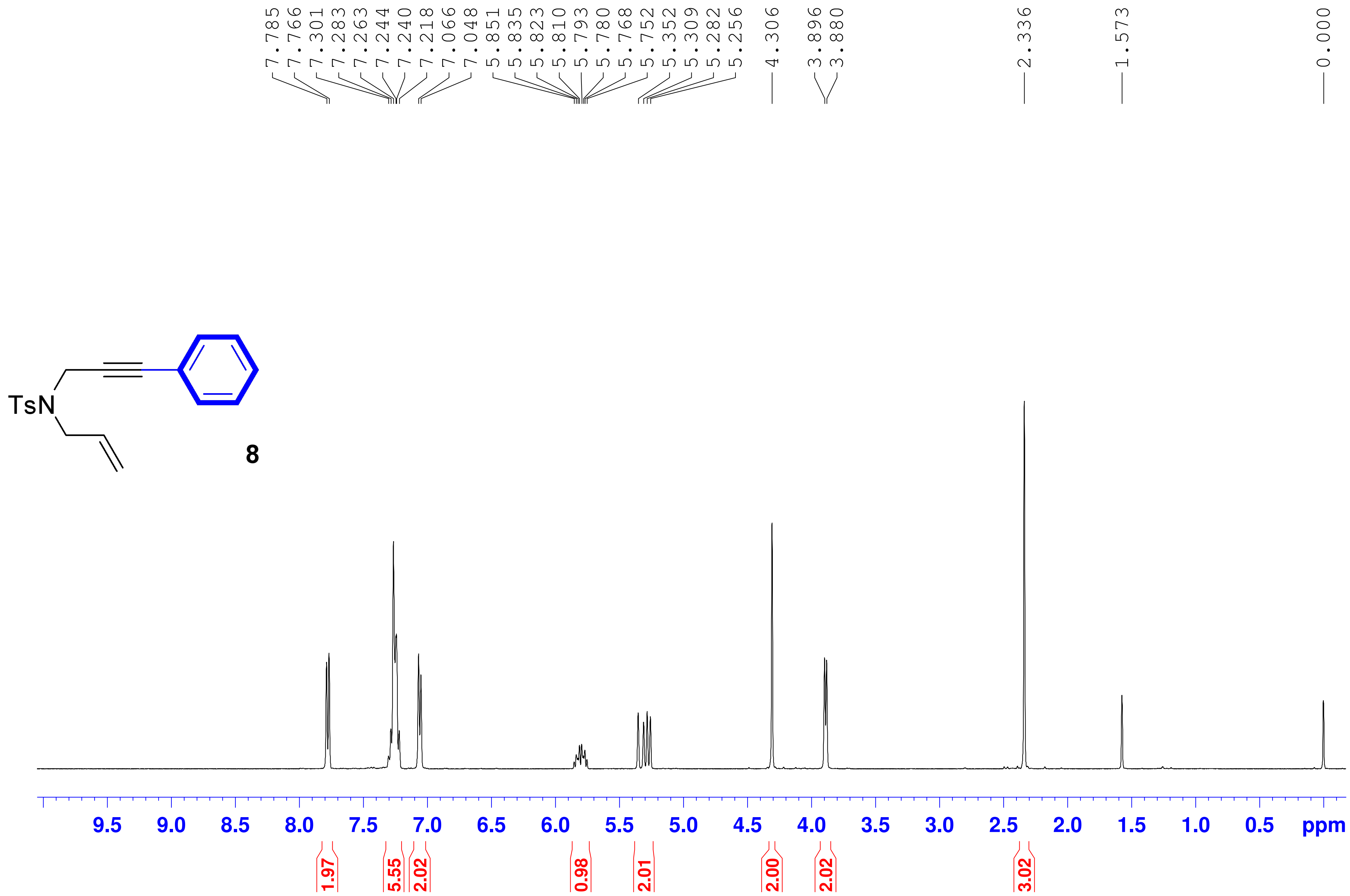

7.785
7.766
7.301
7.283
7.263
7.244
7.240
7.218
7.066
7.048
5.851
5.835
5.823
5.810
5.793
5.780
5.768
5.752
5.352
5.309
5.282
5.256
4.306
3.896
3.880
2.336
1.573
0.000
TsN
8
9.5 9.0 8.5 8.0 7.5 7.0 6.5 6.0 5.5 5.0 4.5 4.0 3.5 3.0 2.5 2.0 1.5 1.0 0.5 ppm
1.97
5.55
2.02
0.98
2.01
2.00
2.02
3.02

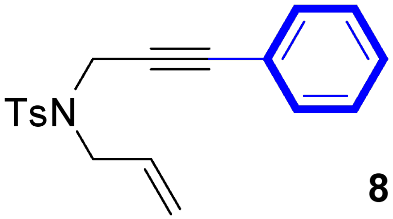
TsN

8

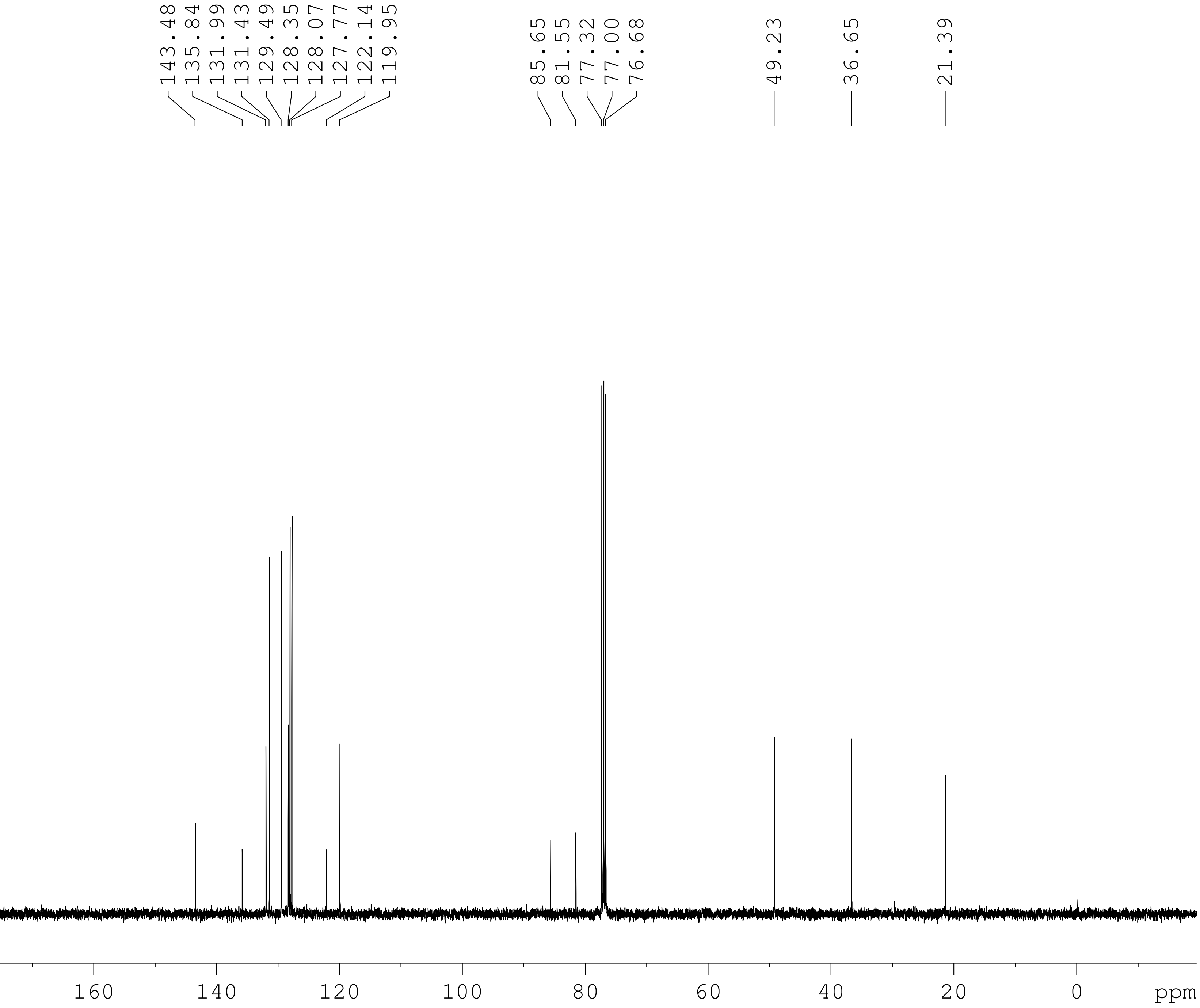
143.48
135.84
131.99
131.43
129.49
128.35
128.07
127.77
122.14
119.95
85.65
81.55
77.32
77.00
76.68
49.23
36.65
21.39
200
180
160
140
120
100
80
60
40
20
0
ppm
